# Leaving the Loners Alone: Dispositional Preference for Solitude Evokes Ostracism

**DOI:** 10.1177/0146167220968612

**Published:** 2020-11-02

**Authors:** Dongning Ren, Anthony M. Evans

**Affiliations:** 1 Tilburg University, The Netherlands

**Keywords:** preference for solitude, ostracism, exclusion, person perception

## Abstract

What are the interpersonal consequences of seeking solitude? Leading theories in developmental research have proposed that having a general preference for solitude may incur significant interpersonal costs, but empirical studies are still lacking. In five studies (total *N* = 1,823), we tested whether target individuals with a higher preference for solitude were at greater risk for ostracism, a common, yet extremely negative, experience. In studies using self-reported experiences (Study 1) and perceptions of others’ experiences (Study 2), individuals with a stronger preference for solitude were more likely to experience ostracism. Moreover, participants were more willing to ostracize targets with a high (vs. low) preference for solitude (Studies 3 and 4). Why do people ostracize solitude-seeking individuals? Participants assumed that interacting with these individuals would be aversive for themselves *and* the targets (Study 5; preregistered). Together, these studies suggest that seeking time alone has important (and potentially harmful) interpersonal consequences.

Solitude is a common experience in everyday life (Larson, 1990). Solitude is defined as the absence of both in person and virtual social interaction, with or without the physical presence of others (e.g., reading alone in a café or from a private room; Burger, 1995; Long et al., 2003). Occasionally, spending time alone may be enjoyable and promote individual well-being (Coplan, Hipson, et al., 2019; Long et al., 2003; Nguyen et al., 2018), but leading theories in developmental research have proposed that having a general preference for solitude may incur significant costs in the interpersonal domain (e.g., peer rejection, being viewed negatively; Coplan, Ooi, et al., 2019). However, these theoretical arguments have received relatively little empirical attention. To contribute to this understudied research area, we examine the interpersonal (i.e., reputational) consequences of dispositional preference for solitude by focusing on a common, yet extremely negative, social outcome: ostracism. Our central hypothesis is that individuals who express a high preference for solitude are at risk of being ostracized by others. Below, we first provide a conceptual overview of preference for solitude—a relatively new construct in the literature, and then turn our attention to the risk of ostracism associated with preference for solitude.

## Dispositional Preference for Solitude

Some people have an appreciation for solitude, finding it to be pleasant, productive, and interesting, whereas others find solitude to be unpleasant, unproductive, and boring. This individual difference is referred to as Preference for Solitude (Burger, 1995; Coplan, Ooi, et al., 2019). More recent work in developmental psychology literature has examined similar constructs in children, such as unsociability (Coplan & Weeks, 2010) and the affinity for aloneness (Goossens, 2014). Arguably, individuals with a high preference for solitude seek solitude because they find time alone pleasant and not because they find social interactions unpleasant. In other words, solitude seekers do not necessarily feel shy or socially anxious (Burger, 1995; Coplan & Armer, 2007; Coplan, Ooi, et al., 2019; Cramer & Lake, 1998). In support of this idea, children with a high preference for solitude do not actively avoid others (Coplan et al., 2004). Similarly, young adults with a high preference for solitude spend more time alone than those with a low preference for solitude. However, most people, regardless of their preference for solitude, consider their time spent with others to be mostly positive (Burger, 1995).

Importantly, preference for solitude is related (but distinct from) the widely researched personality trait of extroversion (Coplan, Ooi, et al., 2019). Preference for solitude is correlated with extroversion as sociability is one of the facets of extroversion (Cattell, 1947; Eysenck & Eysenck, 1964; Hofstee et al., 1992; McCrae & Costa, 1997). However, there are important differences: extroversion is a global trait including multiple facets unrelated to solitude, such as positive affect and assertiveness (Wilt & Revelle, 2016).

### Dispositional Preference for Solitude and Ostracism

We ask whether individuals with a stronger (vs. weaker) preference for solitude are more likely to experience negative interpersonal consequences, specifically, ostracism. We focus on ostracism because it is a common (Nezlek et al., 2012), yet extremely consequential, negative experience (Williams, 2009). Ostracism is defined as being ignored and excluded (Williams, 2009). It can take on a variety of forms ranging from relatively subtle (e.g., denial of eye contact) to overt (e.g., permanent expulsion from groups; Williams, 1997). Critically, being ostracized in its most minimal forms can lead to a variety of negative outcomes, including thwarted need satisfaction (Williams, 2009), negative emotions (Chow et al., 2008), and impaired cognitive functioning (Baumeister et al., 2002).

Why might having a preference for solitude lead to ostracism? Evolutionary theories of stigma argue that, given the importance of coordinated efforts in group living, people have evolved to exclude others who do not conform to familiar interaction norms (Kurzban & Leary, 2001; Neuberg et al., 2000) such as those who are (or perceived to be) socially disengaged (Kerr & Levine, 2008). Similarly, developmental theories propose that children believe solitude seeking violates social norms about peer interactions and therefore respond negatively to peers who choose to be alone (Rubin et al., 1990, 1991). In addition, because solitude seeking—an individual behavior—can be neither threatening nor rewarding interpersonally, a recent theory of interpersonal invisibility (Neel & Lassetter, 2019) suggests that people may deem solitude-seeking others as irrelevant and passively ignore (e.g., overlook) these individuals.

Consistent with these theoretical ideas, preference for solitude is correlated with peer exclusion in children (Coplan et al., 2004; Ladd et al., 2011; Ooi et al., 2018; Spangler & Gazelle, 2009), loneliness in children (Coplan et al., 2013), loneliness (in relationships with peers) in adolescence (Majorano et al., 2015), and ostracism experience in adults (Ren et al., 2016). In addition, children are able to identify those (classmates) who are characterized by a high preference for solitude and neglect these individuals in interpersonal relationships (Harrist et al., 1997). Finally, a few studies examined social perceptions of preference for solitude using experimentally manipulated profiles. In these studies, children reported less interest in becoming friends with a high solitude preference peer (vs. a social peer; Ding et al., 2015; Zava et al., 2020).

However, other studies suggest that preference for solitude may not constitute a risk factor for ostracism. For example, preference for solitude is considered as a benign disposition in emerging adulthood (Bowker et al., 2017; Coplan, Ooi, et al., 2019). In support of this notion, researchers have found that preference for solitude is not associated with qualities of interpersonal relationships (Nelson, 2013), loneliness (Burger, 1995; Long et al., 2003), or social isolation (Waskowic & Cramer, 1999). Furthermore, in a recent study examining perceived acceptability of others’ preference for solitude, participants rated solitude seeking to be highly acceptable (3.61; 1 = *It’s really wrong*, 4 = *It’s perfectly okay*; Bowker et al., 2020). Finally, manipulating extroversion (although not preference for solitude specifically) in a hypothetical target did not affect participants’ ostracism intentions toward the target (Rudert et al., 2020). Given these conflicting findings, it is an open question as to whether solitude-seeking individuals are more likely to experience ostracism.

The present research directly tests the hypothesis that people are more likely to ostracize individuals with high preference for solitude. Williams (1997) proposed that people may use ostracism as a strategy to preemptively avoid any anticipated aversive outcomes—to the self or the targeted individual (defensive ostracism; Williams, 1997; Zadro et al., 2017). Following this line of reasoning, people might be motivated to ostracize individuals with a high preference for solitude for two reasons: One reason is that people may anticipate interacting with a person having a high preference for solitude to be unpleasant for themselves (henceforth referred to as the *self-interested* concern). The other reason is that people assume that social interactions would be unpleasant or undesirable for individuals with a high preference for solitude (henceforth referred to as the *other-regarding* concern). In sum, people may ostracize individuals with a high preference for solitude for either self-interested or other-regarded reasons.

### Current Research

We conducted a series of five studies to examine whether people are more willing to ostracize individuals with a high preference for solitude. First, we estimated the association between preference for solitude and ostracism experience using self-reported experiences and participants’ perceptions of others’ experiences (Studies 1 and 2). Then, we measured participants’ ostracism intentions toward targets with a high (vs. low) preference for solitude (Studies 3 and 4). Finally, we tested the theorized motives (self-interested and other-regarding) as potential mediators (Study 5; preregistered).

In addition to our main research interest in ostracism intentions, we also explored how preference for solitude influences other inferences related to personality and character (Studies 3–5). To this end, participants rated targets on measures assessing social motivation (e.g., the need to belong; Baumeister & Leary, 1995), two universal dimensions of person perception (i.e., warmth and competence; Fiske et al., 2007), and the Big Five personality dimensions. We report these additional results in a separate section after we report all five studies.

All research materials, data, and analysis scripts are available at the Open Science Framework: https://osf.io/s4nez.

## Studies 1 and 2

In the first two studies, we examined the association between preference for solitude and ostracism experience. Past work provides evidence for a zero-order association between the two measures using participants’ self-reported experiences (Ren et al., 2016). Here, we set out to extend this finding in two ways. First, we tested whether this association exists while controlling for related (global) personality traits (Study 1). Because extroversion is related to preference for solitude (Burger, 1995), and at least two dimensions of the Big Five (agreeableness and conscientiousness) affect the risk of ostracism (Hales et al., 2016; Rudert et al., 2020), we measured and included the Big Five scores as covariates. In other words, Study 1 asked whether preference for solitude was correlated with the experience of ostracism even after controlling for global personality traits (i.e., the Big Five).

Second, we moved beyond previous studies, which focused on self-reported measures of ostracism experience, to include peer-report measures of ostracism experience (Study 2). Self-reported ostracism experience is susceptible to the bias of the targets and may not represent the actual experience of being ostracized (Rudert et al., 2020). We thus assessed the ostracism experience by having participants report their evaluations of others’ experiences. We used an acquaintance rating task from the person perception literature (Landy et al., 2016). Each participant rated three targets (a family member, a friend, and an acquaintance) on the two measures of interest: preference for solitude and ostracism experience. As a replication of Study 1, each participant also rated themselves as a fourth target.

Given the methodology similarities between Studies 1 and 2, we present them together in the following section.

### Study 1 Method

#### Participants

Our sample was drawn from a mass prescreening of the undergraduate psychology participant pool at a research university in the Netherlands. The initial sample consisted of 477 participants; four were excluded from data analyses due to missing values, leaving the final sample size of 473 (368 female, 103 male, one other, and one “prefer not to respond”; *M*_age_ = 20.07, *SD* = 3.21, one did not report age). The prescreen survey was available to participants in English and Dutch: 206 completed the survey in English and 267 completed the survey in Dutch. We report aggregated analyses across both language groups, but note that our central results were consistent in analyses looking at each group separately.

#### Procedure and materials

Participants were brought into a laboratory and assigned to individual cubicles to complete a survey packet consisting of several unrelated questionnaires on a computer. Measures of preference for solitude, ostracism experience, and the Big Five traits were embedded in this survey. Participants in the Dutch-language sample completed translated versions of these measures: The Dutch version of the preference for solitude and ostracism experience measures were obtained from Ren & colleagues (2020); the Dutch version of the Big Five measure was obtained from Denissen & colleagues (2008).

#### Dispositional preference for solitude

To measure preference for solitude, we used an adapted version of the Preference for Solitude Scale (Burger, 1995; Ren et al., 2016). The adapted version used similar items from the original scale (16 items; for example, “I need time alone each day.”) but replaced the original forced-choice format with a 7-point scale (1 = *not at all*, 7 = *very much*; α = .89).

#### Ostracism experience

We used the Ostracism Experiences Scale (Carter-Sowell, 2010; Gilman et al., 2013). The scale included eight items rated on a 7-point scale (1 = *hardly ever*, 7 = *almost always*), regarding how often each scenario happens (e.g., “In general, others leave me out of their group”; α = .89).

#### The Big Five

We used the Big Five Inventory (John & Srivastava, 1999). The scale included 44 items in total (e.g., “I see myself as someone who is generally trusting”; 1 = *disagree strongly*, 5 = *agree strongly*). Items were averaged to form an index of each trait: Extroversion (α = .84), Agreeableness (α = .74), Conscientiousness (α = .79), Neuroticism (α = .84), and Openness (α = .78).

### Study 2 Method

#### Participants

We recruited participants on MTurk. Participants were paid US$1.50 and the study took about 12 min to complete. Our planned sample size is based on the recommendation of *n* = 250 or higher for estimating stable correlations (Schönbrodt & Perugini, 2013). Anticipating missing data, we recruited 280 participants (127 female, 153 male; *M*_age_ = 35.52, *SD* = 11.03, range = 19–72).

#### Procedure and materials

Following the procedure of the acquaintance rating task (Landy et al., 2016), each participant evaluated several targets. The original set of targets included targets who are liked or not liked, which may influence participants’ perceptions of the targets’ ostracism experience. To avoid this potential issue, we selected a new set of targets: a family member, a friend, an acquaintance, and the self. For each target, participants were first instructed to visualize the target and enter the target’s initials (this step was omitted for the self); thereafter, participants evaluated target preference for solitude and how often the target was ostracized. Participants always rated themselves after they rated the other three targets; the order of the other three targets was randomized for each participant.

We used the same preference for solitude scale and the ostracism experience scale used in Study 1. The original items were adapted when necessary to reflect other-ratings (e.g., Solitude: “this person needs time alone each day”; Ostracism: “In general, others leave this person out of their group.”). Cronbach’s alphas were calculated for each target separately (Solitude: α_family_ = .95, α_friend_ = .95, α_acquaintance_ = .96, and α_self_ = .95; Ostracism: α_family_ = .97, α_friend_ = .97, α_acquaintance_ = .96, and α_self_ = .97).

For exploratory purposes, we measured participants’ perceptions of the targets’ belonging need (see Supplemental Material, available online, for details). We also included a couple of additional measures that were unrelated to this report (e.g., how well participants knew each target).

### Results

**Figure 1.**
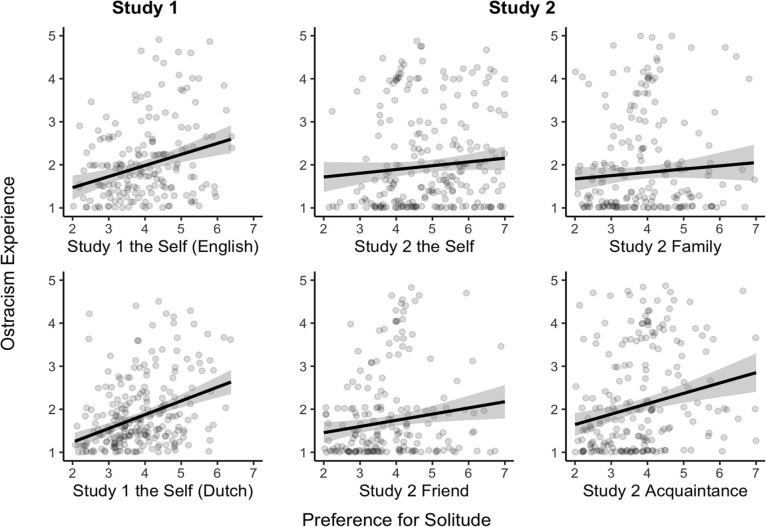
The association between preference for solitude and ostracism experience (Studies 1 and 2).

Visual inspection of the data shows clear evidence for a small-to-medium positive association between preference for solitude and ostracism experience across studies and the rating targets (Figure 1). Zero-order correlations ranged from .16 to .32, *p*s < .008.

#### Study 1

To analyze the data from Study 1, we estimated a regression model with the solitude score as the predictor, the dummy coded language variable (English = 0, Dutch = 1), the big five traits as covariates, and ostracism experience as the outcome variable. We observed a positive association between preference for solitude and ostracism experience: *b* (unstandardized) = 0.11, *p* = .010, 95% confidence interval (CI) = [0.03, 0.19].

#### Study 2

In our second study, observations were clustered within participants and targets. To account for the clustered nature of the data, we estimated a multilevel model with the solitude score (mean-centered within each target) as the predictor and ostracism experience as the outcome variable; random-intercepts were estimated for each participant and each target. We used R packages the lme4 and the lmerTest (Bates et al., 2014; Kuznetsova et al., 2015). We observed a positive association between preference for solitude and ostracism experience, *b* = 0.12, *p* < .001, 95% CI = [0.08, 0.16].

### Discussion

Analyses on participants’ self-ratings (while controlling for the Big Five scores) and their ratings of others converged to demonstrate that higher preference for solitude was robustly associated with more ostracism experience. One limitation of these studies is the correlational nature of the data. Building on these results, in the next two studies we experimentally manipulated participants’ perception of preference for solitude in targets.

## Studies 3 and 4

Our next studies measured ostracism intentions toward targets with experimentally manipulated levels of preference for solitude (Study 3: low, high; Study 4: low, average, and high). In both studies, participants were randomly assigned to evaluate a hypothetical target. The target’s preference for solitude was manipulated using verbal descriptions (Study 3) and simulated personality scale responses (Study 4). Thereafter, participants indicated their intention to ostracize the target. Given the methodology similarities between Studies 3 and 4, we present them together in the following section.

### Study 3 Method

#### Participants

Introductory psychology students at a research university in the United States participated in our study for course credits. Power analysis indicated a minimum sample size of 128 (Cohen’s *d* = 0.5, 80% power, *p* = .05, two-tailed). Using 128 as a guideline, we collected data for 1 week and were able to obtain 142 participants (77 female, 65 male; *M*_age_ = 19.27 years, *SD* = 1.18).

#### Procedure and materials

Participants were brought into a laboratory and assigned to individual cubicles to complete the study on a computer. Participants received a description of a fellow student who expressed either a low (*n* = 71) or a high (*n* = 71) preference for solitude (e.g., “I am the kind of person who doesn’t prefer being alone.” vs. “I am the kind of person who prefers being alone.”). The descriptions were based on the items from the preference for solitude scale we used in Study 1 (Ren et al., 2016). Importantly, aside from preference for solitude (low vs. high), the descriptions of the target were held constant across conditions (e.g., enjoying watching movies and good food; see Supplemental Material, available online, for the descriptions).

After reading this description, participants indicated their ostracism intentions toward the target (eight items, α = .87; for example, “I would give this person little attention in a group.” 1 = *not at all*, 5 = *very much*) and completed one manipulation check item (“This person prefers solitude.” 1 = *strongly disagree*, 5 = *strongly agree*). The ostracism intention items are highly similar to the signals of exclusion (specifically, avoiding and disengaging) summarized in Kerr and Levine (2008) and the items used in past studies (e.g., Rudert et al., 2020; Wirth et al., 2020).

Participants also rated their perceptions of the target on several measures. These measures were further included in Studies 4/5. We report these results from all three studies in the “Additional analyses: Perceptions of targets” section (for brevity, we will not repeat this information in the “Method” section of Studies 4 and 5).

### Study 4 Method

#### Participants

Introductory psychology students at a research university in the United States participated in our study for course credits. We started data collection at the end of one semester and collected data as much as possible in that semester. We were able to get 144 participants. Eight participants were excluded from analyses due to attention-check failure. The final sample consisted of 136 participants (54 female, 81 male; *M*_age_ = 19.22 years, *SD* = 1.44; one did not report gender or age).

#### Procedure and materials

We used the same procedure and dependent measures as in Study 3. The only departures from Study 3 were the manipulation method and the addition of the average condition. Participants were randomly assigned to receive one of three target profiles (preference for solitude: low *n* = 44, average *n* = 43, and high *n* = 49): a copy of a completed questionnaire, ostensibly filled in by a fellow student—the target. The questionnaire was the preference for solitude scale that we used in Study 1 (Ren et al., 2016). The items and the hypothetical target’s responses to each item using Likert-type scales were presented; to avoid demand effects, the title of the questionnaire was not revealed. Participants were instructed to review the completed questionnaire and form an impression of the target.

To create the target profiles, we drew a sample from mass prescreening of the undergraduate psychology participant pool at the same university (*N* = 799; 317 male, 482 female; *M*_age_ = 19.18 years, *SD* = 2.42). Participants completed a packet of survey in an online session. The preference for solitude scale we used in Study 1 was embedded in this survey (other measures are irrelevant to this report and thus are not being discussed here). We computed a composite solitude score for each participant and subsequently created two subsamples, consisting of participants who scored 1 *SD* below the mean (Subsample 1: *n* = 132) or above the mean (Subsample 2: *n* = 112). We then calculated the average score for each item of the scale in both subsamples and the entire sample. Following the three sets of averaged item scores (Subsample 1, the entire sample, and Subsample 2; see Supplemental Material, available online, for the item scores), we filled in the solitude questionnaire to create three targets with varying levels of solitude preferences (low, average, and high). Images of the completed solitude questionnaires were presented to participants as manipulation materials (see Supplemental Material available online).

Finally, participants completed the same measures from Study 3: ostracism intentions (α = .88) and the manipulation check item.

### Results

#### Study 3

We estimated a regression model with the dummy coded condition variable (low preference for solitude as the reference category) as the only predictor and the manipulation check item as the dependent variable. Our manipulation was successful. As intended, participants in the high preference for solitude condition rated the target higher on preference for solitude than those in the low preference for solitude condition, *b* = 3.04, *p* < .001, 95% CI = [2.75, 3.33].

**Figure 2.**
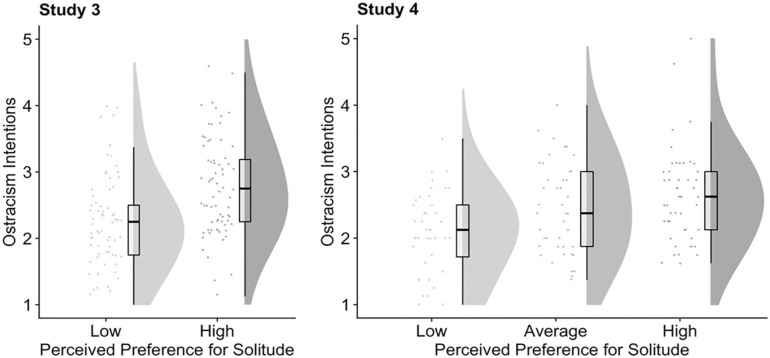
The effects of perceived preference for solitude in targets (Studies 3 and 4).

We then estimated a similar regression model for participants’ ostracism intentions. As predicted, participants reported stronger ostracism intentions for the high (vs. low) solitude preference target, *b* = 0.55, *p* < .001, 95% CI = [0.32, 0.79]. These results are illustrated in Figure 2 (left panel) using raincloud plots, a recently introduced tool for robust data visualization (Allen et al., 2019). Each plot below consists of three components (from left to right): raw data points, a boxplot displaying sample median and interquartile range, and a half violin plot illustrating the data distribution.

Finally, given that men and women may hold different beliefs about solitude (Bowker et al., 2020), we explored the potential effects of observer (participant) gender in this study (and in Studies 4 and 5). No reliable gender effects were obtained; see Supplemental Material, available online, for details.

#### Study 4

Using the same analytic approach as in Study 3, we estimated regression models with the dummy coded condition variables as predictors. Reference categories varied across models to allow for the relevant comparisons and were specified for each effect below. Our manipulation was successful. As intended, we observed a stepwise increase in participants’ ratings of preference for solitude as the target’s preference for solitude increased from low to average, *b* = 0.91, *p* < .001, 95% CI = [0.50, 1.32] (ref: low), and average to high, *b* = 0.88, *p* < .001, 95% CI = [0.49, 1.28] (ref: average).

Turning to ostracism intentions, we observed a similar stepwise pattern of increase but the increase was nonsignificant when comparing the high preference for solitude target with the average target: from low to average, *b* = 0.38, *p* = .008, 95% CI = [0.10, 0.66] (ref: low), and from average to high, *b* = 0.17, *p* = .233, 95% CI = [–0.11, 0.44] (ref: average). See Figure 2 (right panel).

### Discussion

Across two experiments using verbal descriptions (Study 3) and simulated personality data (Study 4), we established that people are more willing to ostracize target individuals who have a high (vs. low) preference for solitude. Notably, people are generally reluctant to ostracize others, indicated by the majority of the responses staying lower than the scale midpoint in both studies (see Figure 2). This reluctance has been observed in past studies that used similar designs (Hales et al., 2016; Rudert et al., 2020). One possible reason for this reluctance could be that inclusion is generally the norm in social interactions (Rudert & Greifeneder, 2016), and people do not deliberately use ostracism unless it is justified (Sommer & Yoon, 2013). Nonetheless, we obtained consistent evidence that people are more likely to ostracize targets with higher preference for solitude.

## Study 5

Why are people more willing to ostracize solitude seekers? Ostracism intentions might be motivated by both self-interested and other-regarded reasons. People may anticipate that interacting with the target would not be enjoyable (the self-interested concern), and thus preemptively exclude the target to avoid any aversive outcomes to themselves. People may also assume that the target would not find social interactions enjoyable (the other-regarding concern), and thus exclude the target to avoid any aversive outcomes to the target. In this study, we measured both concerns, tested whether our manipulation of solitude preference influence these concerns, and whether these concerns explain the effect of the manipulation on ostracism intentions.

We used the manipulation method of Study 4. We created two new targets profiles (preference for solitude: low vs. high) based on a sample drawn from the university where we conducted this study. The local profiles were used, given there could be potential cultural differences in preference for solitude between the two countries where we collected data, namely, from the United States (Study 4) and the Netherlands (Study 5).

Measures, data collection, and analyses were preregistered: https://aspredicted.org/blind.php?x=sy4u9q.

### Method

#### Participants

Introductory psychology students at a research university in the Netherlands participated in this study for course credits. Our sample size was determined by (a) a power analysis on our main dependent variable of ostracism intentions, and (b) the standard laboratory procedure at the university. The previous study (Study 4) used a similar manipulation method and produced a medium effect (Cohen’s *d* = 0.6). To detect a medium effect (Cohen’s *d* = 0.5, 80% power, α = .05, two-tailed), the minimum sample size would be 128 participants. Anticipating data exclusions due to missing values and attention-check failures, we planned to recruit at least 150 participants. Following the standard data collection procedure at the university, we collected data for 2 weeks, and a total number of 800 students completed the study. Eight participants were excluded from the analyses for failing both attention-check items. The final sample consisted of 792 participants (628 female, 158 male, and five “other”; *M*_age_ = 20.08 years, *SD* = 2.71; one participant did not report gender or age).

#### Procedure and design

Participants completed this study online. The procedure was identical to Study 4: participants received a copy of a completed preference for solitude scale ostensibly filled in by a fellow student (preference for solitude: low *n* = 395, high *n* = 397). They were instructed to review the scale and form an impression.

To create the target profiles, we used the sample from Study 1: a sample of college students at the same university. Following the steps in Study 4, we prepared images of two completed solitude questionnaires (low vs. high) based on a subset of the sample who scored 1 *SD* below the mean (*n* = 74) and a subset of the sample who scored 1 *SD* above the mean (*n* = 81). See Supplemental Material, available online, for the item scores and the images.

#### Outcome measures

All items were rated on the same 5-point scale (1 = *not at all*, 5 = *very much*).

#### Self-interested and other-regarding concerns

We measured the self-interested concern using three items (e.g., “I would probably not have a good time hanging out with this person at social events”; α = .82), and the other-regarding concern using three items (e.g., “This person would probably not have a good time at social events”; α = .90). The order of the two measures was random for each participant.

#### Ostracism intentions

We used a brief version of the measure we used in Studies 3 and 4. This brief version has four items with slightly improved wordings to encourage participants to endorse the items (α = .72). For example, the original item “I would give this person little attention in a group” was adapted to “I might find myself giving this person little attention in a group.”

### Results

**Figure 3.**
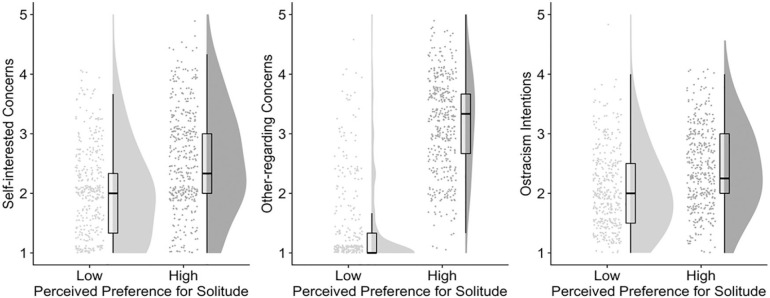
The effects of perceived preference for solitude in targets (Study 5).

We first tested the effects of the manipulation on the outcome variables. For each outcome variable, we estimated a regression model with the dummy coded manipulation (low as the reference category) as the predictor. Perceiving high (vs. low) solitude preference in the target increased participants’ self-interested concerns, *b* = 0.56, *p* < .001, 95% CI = [0.45, 0.68], other-regarding concerns, *b* = 1.77, *p* < .001, 95% CI = [1.66, 1.88], and ostracism intentions, *b* = 0.37, *p* < .001, 95% CI = [0.28, 0.46]. Results are illustrated in Figure 3.

**Figure 4.**
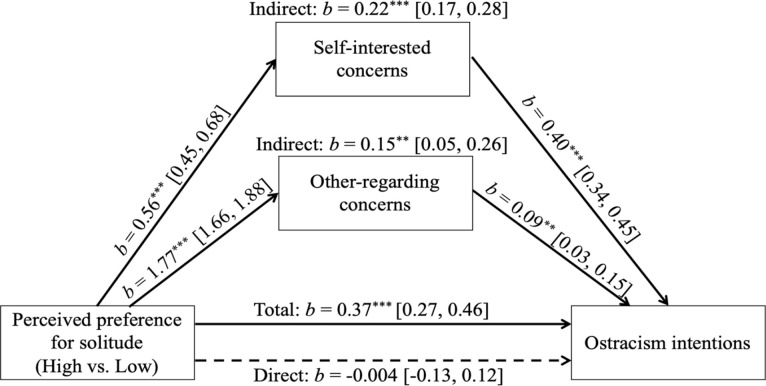
Multiple mediation model (Study 5). *Note. b*s are unstandardized. 95% confidence intervals are in brackets. Solid lines indicate significant paths; dashed lines indicate nonsignificant paths. *p < .05. **p < .01. ***p < .001.

Next, we conducted a multiple mediation model, testing both concerns as simultaneous mediators (Preacher & Hayes, 2008). We used the lavaan package in R (Rosseel, 2012) and requested the bias-corrected and accelerated bootstrap intervals based on 5,000 samples. Both indirect effects had CIs that did not contain 0 (Figure 4). The magnitude of the two indirect effects did not differ, *b* = 0.07, *p* = .275, 95% CI = [–0.06, 0.20].

We further inspected individual paths in the mediation model (Yzerbyt et al., 2018). Interestingly, whereas the magnitude of the alpha path estimate for self-interested concerns (vs. other-regarding concerns) was only about one third (0.56 vs. 1.77, *b* = −1.21, *p* < .001, 95% CI = [−1.33, −1.08]), the magnitude of *b* path estimate for self-interested concerns (vs. other-regarding concerns) was about 4 times as large (0.40 vs. 0.09, *b* = 0.31, *p* < .001, 95% CI = [0.21, 0.41]), indicating that self-interested (vs. other-regarding) concerns were a stronger predictor of ostracism intentions toward the target.

### Discussion

The results of our final study suggest that ostracism intentions toward targets with higher preference for solitude are motivated by both self-interested and other-regarded concerns. Interestingly, although people have stronger other-regarding (vs. self-interested) concerns for such a target, self-interested (vs. other-regarding) concerns are a stronger motive behind their ostracism intentions.

## Additional Analyses: Perceptions of Targets

In our main analyses, we presented evidence that people are more willing to ostracize solitude-seeking individuals. Here we ask a related yet different question: How do people perceive solitude-seeking individuals? We explored this question in Studies 3 to 5. Participants rated the targets on measures assessing social motivation; warmth and competence, the two person perception dimensions; and the Big Five personality dimensions.

### Measures

#### Perceived social motivation

We used two indicators to assess perceived social motivation of the targets: perceived belonging need and anticipated reactions to belonging events. Perceived belonging need was measured using the single-item need to belong scale (Nichols & Webster, 2013; adapted to reflect other-ratings: “This person has a strong need to belong”; 1 = *strongly disagree*, 5 = *strongly agree*). This measure was included in Studies 3 and 4. Anticipated reactions to belonging events was measured using eight items in Study 3 (α = .97; for example, “This person would be bothered a great deal when they are not included in other people’s plans”; 1 = *strongly disagree*, 5 = *strongly agree*) and a brief version in Study 4 (four items; α = .82).

#### Perceived warmth and competence

We measured how the targets were perceived on the dimension of warmth (kind, warm, and friendly; αs = .90, .92, and .86) and competence (competent, intelligent, and smart; αs = .82, .83, and .80) in Studies 3 to 5. The items were presented in a randomized order for each participant. We used a 7-point scale (1 = *not at all*, 7 = *very much*) in Studies 3 and 4, and a 5-point scale (1 = *not at all*, 5 = *very much*) in Study 5. Because other outcome variables used a 5-point scale in all studies, in Studies 3 and 4 we rescaled these two variables from 1 to 7 to range from 1 to 5 before analyzing them, so that the means of all outcome variables and the estimates of all models are comparable.

#### Perceived traits

We adapted the items from the Big Five Inventory (used in Study 1; John & Srivastava, 1999) to measure how the targets were perceived on the five dimensions of personality in Studies 3 to 5 (e.g., “I see this person as someone who is generally trusting”: 1 = *disagree strongly*, 5 = *agree strongly*): extroversion (Studies 3–5, αs = .97, .96, and .96), agreeableness (αs = .85, .82, and .70), conscientiousness (αs = .70, .75, and .75), neuroticism (α = .83, .80, and .82), and openness (αs = .70, .67, and .63).

### Analysis Plan

We analyzed each study separately. For perceived belonging need and anticipated reactions to belonging events, we estimated regression models with the dummy coded condition variable(s) as the predictor(s), with the low preference for solitude condition as the reference category. For each person perception dimension (warmth and competence), we estimated regression models with the dummy coded condition variable(s) as the predictor(s), and the other dimension as a covariate. Similarly, for each personality trait (outcome variable), we estimated regression models with the dummy coded condition variable(s) as the predictor(s), and the other four traits as covariates.

### Results

**Figure 5.**
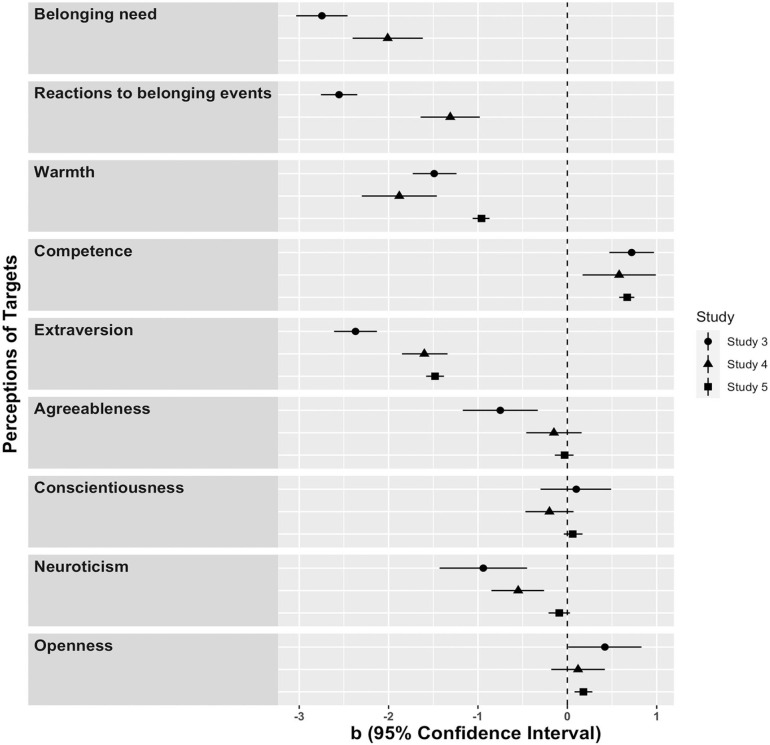
Perceptions of targets (Studies 3–5).

Here, we focus on the effects of having a high (vs. low) preference for solitude—the effect we examined consistently across studies (additional effects are reported in Supplemental Material, available online). The unstandardized regression coefficients with their associated 95% CIs for Studies 3 to 5 are plotted in Figure 5. The effects of target preference for solitude on agreeableness, conscientiousness, neuroticism, and openness were inconsistent across studies. Five findings remained robust: Targets with a high (vs. low) preference for solitude were judged to be lower in need to belong, less reactive to belonging events, less warm, more competent, and less extroverted. We will return to these findings in the “General Discussion” section.

## General Discussion

Across five studies, we found consistent evidence that individuals who voluntarily seek solitude are at greater risk for ostracism. This conclusion is based on correlational evidence, using participants’ self-reported experiences and their perceptions of others’ experiences (Studies 1 and 2), as well as experimental evidence using verbal descriptions and simulated personality profiles (Studies 3–5). These findings were robust across the contexts of data collection: the United States and the Netherlands; online and in a laboratory; from college students and MTurk.

Importantly, our final study provides some insight into why people ostracize targets with higher preference for solitude. Ostracism intentions are related to both self-interested *and* other-regarding motives (Study 5). This finding supports and builds on Williams’ theorizing that people may use ostracism preemptively to avoid any aversive outcomes (Williams, 1997). Comparing the two motives further revealed that self-interest, wanting to avoid an unpleasant social interaction, was the primary motive underlying participants’ ostracizing intentions.

Our work also provides insights into general beliefs about solitude-seeking individuals (Studies 3–5): participants considered solitude-seeking individuals to be low in the need to belong, indifferent to belonging events, cold, competent, and introverted. While most of these evaluations are intuitive, given the conceptual link between preference for solitude and low sociality, the positive relationship between preference for solitude and competence is surprising. Past studies found that people believe that loneliness and introversion are associated with incompetence (Anderson & Kilduff, 2009; Lau & Gruen, 1992), suggesting that lay people are able to distinguish preference for solitude from loneliness and introversion. Why do people perceive high (vs. low) solitude preference targets to be more competent? One possible interpretation is that preference for solitude is perceived as a sign of maturity, given that as people transition from adolescent to adulthood, choosing to spend time in solitude becomes more normative and purposeful (Coplan, Ooi, et al., 2019). Another possible interpretation is that preference for solitude is linked with independence in lay beliefs. In fact, wanting to be alone is termed as a motivation for independence in the fundamental social motives framework (Neel et al., 2016). Future research should try to better understand the intriguing link between preference for solitude and competence in lay beliefs.

### Theoretical Contributions

The current research contributes to the growing literature on voluntary solitude. To date, there is a general lack of studies on the voluntary preference for solitude, and there are even fewer studies using adult samples or providing causal evidence (Coplan, Ooi, et al., 2019). Our research contributes to this literature by presenting clear evidence that having a strong preference for solitude is consequential in the interpersonal domain. The desire for “me time” is commonly experienced (e.g., Larson, 1990), and there are many potential benefits that voluntary solitude affords (e.g., Long et al., 2003). However, our research sheds light on potential barriers (and consequences) to seeking solitude—the risk of being ostracized and stigmatized.

The current studies suggest that the link between preference for solitude and ostracism could be dynamic and recursive. Targets of ostracism may withdraw from social interactions to minimize risk of additional social pain (Richman & Leary, 2009; Van Kleef et al., 2010). In past experiments, targets of ostracism (vs. inclusion) indicated stronger intentions to disengage from social situations (Pfundmair et al., 2015), more positive ratings of physical spaces that hinder social interaction (Meagher & Marsh, 2017), and, importantly, a higher preference for being alone in the following activity (Ren et al., 2020). Here, we showed that, ironically, the very response to ostracism (i.e., preference for solitude) may put targets at higher risk for ostracism in future social interactions. To fully establish this bidirectional causal link between preference for solitude and ostracism, future work should track participants longitudinally.

The current studies also broaden our understanding of *who* is ostracized. Focusing on the broad Big Five dimensions, past studies identified two risk factors: low agreeableness and low conscientiousness (Rudert et al., 2020). Notably, narrow traits are often able to better predict domain-specific behavioral outcomes, even when controlling for global traits (Dudley et al., 2006; Paunonen et al., 2003). Here, we focus on preference for solitude, a narrow, domain-specific trait, as both preference for solitude and ostracism are conceptually related to absence of social interactions (although in the case of ostracism, the absence is involuntary). We found that preference for solitude was associated with general ostracism experience, even while controlling for the Big Five traits (Study 1); in addition, participants did not consistently infer agreeableness or conscientiousness from targets’ preference for solitude (Studies 3–5: analyses on perceptions of targets). Taken together, these findings demonstrated that a narrow trait—preference for solitude—put individuals at heightened risk for ostracism above and beyond the known dispositional factors of agreeableness and conscientiousness. An interesting direction for future research is to explore other narrow, domain-specific traits (e.g., trait aggression) and examine multiple risk factors for ostracism in one study. This would allow researchers to examine the relative importance of each risk factor and any potential interaction effects between these factors.

More broadly, the current studies shed light on the question of *why* people ostracize others. Empirical attention has been given to self-interested or malicious reasons such as using ostracism to punish deviant or burdensome behaviors (Schachter, 1951; Wesselmann et al., 2013; Wirth et al., 2020). Our research adds to this literature by showing that people may have self-interested and other-regarding reasons for ostracizing others. This other-regarding motive for ostracism is in fact not uncommon in our daily life: people may stay silent during an argument with their partner to avoid saying anything harmful, refrain from inviting a busy coworker out for drinks so as not to distract them, or withhold information from a friend when they believe the information may hurt their feelings (a form of partial ostracism; Jones & Kelly, 2013). All these behaviors, albeit motivated by genuine concerns for the target individual, are still examples of the act of ostracizing.

### Limitations and Additional Future Directions

In Studies 3 to 5, we used hypothetical profiles to manipulate preference for solitude. This approach is limited in two ways. First, the profiles (e.g., the verbal descriptions in Study 3) may not represent the actual levels of preference for solitude of individuals people encounter in their social environment. Recognizing this potential issue, in Studies 4 and 5, we used a data-driven approach of generating the hypothetical profiles. Second, the profiles made the information of preference for solitude explicit to the participants. In real life, people sometimes indeed make interpersonal decisions based on explicit personality information, for instance, in the domains of personnel selection (Ones & Viswesvaran, 1996) and romantic partner choice (Hall et al., 2010). Yet, at other times, people lack explicit knowledge of their interaction partners. Is preference for solitude a visible trait in social interaction? In other words, can people accurately infer others’ preference for solitude?

Past studies have not examined this question directly. However, there is suggestive evidence that people readily detect the preference for solitude in others. For example, children are able to recognize their peers’ preference for solitude and interact with these individuals accordingly (e.g., overlook them; Harrist et al., 1997). Similarly, adult participants can detect their friends’ motivation to spend time alone (referred to as independence) with some accuracy (Huelsnitz et al., 2020). Generally, people accurately detect personality traits in a target person based on brief interactions or minimal information (Connelly & Ones, 2010; Tskhay & Rule, 2014). Moreover, compared with other traits, extroversion (a related construct) is more visible and more accurately rated by perceivers (Connelly & Ones, 2010).

Another limitation in our experiments is that we measured participants’ ostracism intentions; yet intentions do not necessarily predict actual behavior (e.g., Ajzen, 1991). However, we speculate that there is a relatively strong link between ostracism intentions and behavior (vs. other active forms of exclusion such as physical aggression; Kerr & Levine, 2008). Ostracism does not require an action (Williams, 1997, 2009). In fact, it may take minimal effort to engage in ostracizing (e.g., not saying hello; Kerr & Levine, 2008). In addition, the ambiguous nature of ostracism makes it hard to be documented and thus sources may not be held accountable. It has been shown that ostracism (vs. harassment) is perceived to be more socially acceptable and less regulated at the workplace (O’Reilly et al., 2015). Finally, people tend to underestimate others’ social sufferings caused by ostracism (Nordgren et al., 2011), suggesting that the act of ostracizing is believed to be relatively inconsequential, which may further contribute to the link between intentions and behavior.

In addition to the limitations of the experiments, we collected data from Western countries (the United States and the Netherlands) in all five studies. This puts constraints on the generalizability of the results to other cultural contexts. It has been observed that people in Western cultures are more encouraged to be sociable and expressive, whereas people from East Asian cultures are more encouraged to be shy and self-reflective (Chen, 2010; Ding et al., 2015; Oyserman et al., 2002). Consistent with this observation, past research has suggested that solitude is more valued and experienced more positively in East Asian cultures than in Western cultures (Jiang et al., 2019). Thus, solitude-seeking individuals might be perceived more positively and at less risk for ostracism in East Asian cultures versus Western cultures. These ideas point to a fruitful avenue for future research.

Finally, future research should examine whether or not people’s judgments of those who prefer solitude are accurate. Participants in our studies assumed that preference for solitude is an undesirable disposition in social interactions. They anticipated interactions to be unpleasant for themselves and for the target individual. But are these valid concerns? We speculate that people might over-rely on preference for solitude as a predictor of social interaction outcomes. Because preference for solitude is not an indicator of a lack of interest in social interactions (Coplan, Ooi, et al., 2019), the need to affiliate is a basic need that applies to everyone (Baumeister & Leary, 1995), and that the immediate impact of ostracism is universally aversive regardless of dispositional characteristics of the targeted individual (McDonald & Donnellan, 2012), it is likely that individuals with a high preference for solitude would enjoy social interactions as much as others. Dispelling these misconceptions of solitude-seeking individuals might be an effective strategy to promote inclusive behaviors.

## Conclusion

The research on preference for solitude is still at its infancy stage. Our research advances our understanding of the interpersonal consequences of having a general preference for solitude. Our findings suggest that, seeking time alone—an essentially individual behavior that may afford many potential benefits—comes at the cost of social inclusion.

## Supplemental Material

Ren_Online_Appendix – Supplemental material for Leaving the Loners Alone: Dispositional Preference for Solitude Evokes Ostracism Click here for additional data file. Supplemental material, Ren_Online_Appendix for Leaving the Loners Alone: Dispositional Preference for Solitude Evokes Ostracism by Dongning Ren and Anthony M. Evans in Personality and Social Psychology Bulletin

Supplementary_Materials_2020_08_30 – Supplemental material for Leaving the Loners Alone: Dispositional Preference for Solitude Evokes Ostracism Click here for additional data file. Supplemental material, Supplementary_Materials_2020_08_30 for Leaving the Loners Alone: Dispositional Preference for Solitude Evokes Ostracism by Dongning Ren and Anthony M. Evans in Personality and Social Psychology Bulletin
