# Supplementary material for: Leaving the Loners Alone: Dispositional Preference for Solitude Evokes Ostracism
Source: Pers Soc Psychol Bull. 2020 Nov 2;47(8):1294–308. doi: 10.1177/0146167220968612 (PMC8258721; doi:10.1177/0146167220968612)
Supplement: Ren_Online_Appendix – Supplemental material for Leaving the Loners Alone: Dispositional Preference for Solitude Evokes Ostracism [file Ren_Online_Appendix.docx]

Table of contents

[Study 1 2](#_Toc39066117)

[Preference for solitude (English) 2](#_Toc39066118)

[Preference for solitude (Dutch) 2](#_Toc39066119)

[Ostracism experiences (English) 3](#_Toc39066120)

[Ostracism experiences (Dutch) 3](#_Toc39066121)

[Big Five Inventory (English) 4](#_Toc39066122)

[Big Five Inventory (Dutch) 5](#_Toc39066123)

[Demographics (English) 6](#_Toc39066124)

[Demographics (Dutch) 6](#_Toc39066125)

[Study 2 7](#_Toc39066126)

[Peer ratings: Preference for solitude 7](#_Toc39066127)

[Peer ratings: Ostracism experiences 8](#_Toc39066128)

[Peer ratings: The need to belong 8](#_Toc39066129)

[Self ratings: Preference for solitude 8](#_Toc39066130)

[Self ratings: Ostracism experiences 9](#_Toc39066131)

[Self ratings: The need to belong 9](#_Toc39066132)

[Demographics 9](#_Toc39066133)

[Study 3 10](#_Toc39066134)

[Manipulation 10](#_Toc39066135)

[The Big Five 10](#_Toc39066136)

[Person perception 12](#_Toc39066137)

[The need to belong 12](#_Toc39066138)

[Reactions to belonging events 12](#_Toc39066139)

[Ostracism intentions 12](#_Toc39066140)

[Manipulation check 13](#_Toc39066141)

[Demographics 13](#_Toc39066142)

[Study 4 14](#_Toc39066143)

[Manipulation 14](#_Toc39066144)

[The Big Five 15](#_Toc39066145)

[Person perception 17](#_Toc39066146)

[The need to belong 17](#_Toc39066147)

[Reactions to belonging events 17](#_Toc39066148)

[Ostracism Intentions 17](#_Toc39066149)

[Manipulation check 18](#_Toc39066150)

[Demographics 18](#_Toc39066151)

[Study 5 19](#_Toc39066152)

[Manipulation 19](#_Toc39066153)

[Self-interested concerns 20](#_Toc39066154)

[Other-regarding concerns 20](#_Toc39066155)

[Ostracism intentions 21](#_Toc39066156)

[Person perception 21](#_Toc39066157)

[The Big Five 21](#_Toc39066158)

[Demographics 22](#_Toc39066159)

# Study 1

## Preference for solitude (English)

To what extent does each of the following statements apply to you? Please respond using a 7-point scale:

| 1 | 2 | 3 | 4 | 5 | 6 | 7 |
| --- | --- | --- | --- | --- | --- | --- |
| Not at all |  |  |  |  |  | Very much |

I do not like being alone.

Being apart from other people for long periods of time does not bother me.

I enjoy the pleasure of solitude.

I see myself as a loner.

The perfect weekend is spent alone.

I prefer spending Friday night alone rather than being with others.

The perfect vacation would be with lots of people around.

My dream vacation is to be alone with no connections to others.

With free time, I prefer to be with others.

I feel I can think clearer when alone.

Solitude helps me center myself.

I do not understand people who choose to be alone.

I need time each day alone to collect my thoughts.

Spending time alone enhances my day.

I need time alone each day.

Being with others for extended amounts of time becomes unbearable.

## Preference for solitude (Dutch)

Geef aan in hoeverre je het eens of oneens bent met de volgende stellingen op een schaal:

| 1 | 2 | 3 | 4 | 5 | 6 | 7 |
| --- | --- | --- | --- | --- | --- | --- |
| helemaal niet |  |  |  |  |  | heel erg |

Ik hou er niet van om alleen te zijn.

Ik vind het niet erg om voor een lange periode gescheiden te zijn van andere mensen.

Ik vind het fijn om alleen te zijn.

Ik zie mezelf als een einzelgänger.

Het perfecte weekend is een weekend alleen.

Ik ben liever een vrijdagavond alleen dan met anderen.

De perfecte vakantie is met veel mensen om me heen.

Mijn droomvakantie is om alleen te zijn zonder contact met anderen.

In mijn vrije tijd ben ik het liefst met andere mensen.

Ik heb het gevoel dat ik helderder kan denken als ik alleen ben.

Alleen zijn helpt me om mijn hoofd leeg te maken.

Ik snap niet waarom mensen ervoor kiezen om alleen te zijn.

Ik heb elke dag tijd nodig om alleen te zijn, zodat ik mijn gedachten op een rijtje kan zetten.

Tijd alleen doorbrengen maakt mijn dag beter.

Ik heb elke dag tijd alleen nodig.

Langere tijd met anderen zijn wordt onverdraaglijk.

## Ostracism experiences (English)

For each of the following statements, please consider your personal feelings. Determine how often, in general, the following experiences happen to you. Just give your gut response. Use the sale provided:

| 1 | 2 | 3 | 4 | 5 | 6 | 7 |
| --- | --- | --- | --- | --- | --- | --- |
| Hardly ever |  |  | Sometimes |  |  | Almost always |

In general, others leave me out of their group.

In general, others keep me out-of-the-loop on information.

In general, others treat me as if I am invisible.

In general, others give me the cold shoulder treatment.

In general, others physically turn their backs to me when in my presence.

In general, others treat me as if I’m in solitary confinement.

In general, others do not look at me when I’m in their presence.

In general, others ignore me during their conversation.

## Ostracism experiences (Dutch)

Denk bij elk van de volgende stellingen aan je eigen gevoelens. Geef aan hoe vaak, over het algemeen, je de volgende ervaringen hebt. Geef gewoon je eerste intuïtieve reactie.

| 1 | 2 | 3 | 4 | 5 | 6 | 7 |
| --- | --- | --- | --- | --- | --- | --- |
| Bijna nooit |  |  | Soms |  |  | Bijna altijd |

In het algemeen houden anderen me buiten hun groep.

In het algemeen weerhouden anderen informatie van me.

In het algemeen behandelen anderen me alsof ik onzichtbaar ben.

In het algemeen keren anderen mij de rug toe.

In het algemeen keren mensen mij letterlijk de rug toe als ik aanwezig ben.

In het algemeen behandelen anderen me alsof ik in eenzame opsluiting zit.

In het algemeen kijken anderen niet naar me als ik aanwezig ben.

In het algemeen negeren anderen me tijdens hun gesprek.

## Big Five Inventory (English)

Here are a number of characteristics that may or may not apply to you. For example, do you agree that you are someone who likes to spend time with others? Please choose the number next to each statement to indicate the extent to which you agree or disagree with that statement. Your responses are completely confidential.

| 1 | 2 | 3 | 4 | 5 |
| --- | --- | --- | --- | --- |
| Disagree  Strongly | Disagree  a little | Neither agree  nor disagree | Agree  a little | Agree  strongly |

I am someone who…
1.     Is talkative
2.     Tends to find fault with others
3.     Does a thorough job
4.     Is depressed, blue
5.     Is original, comes up with new ideas
6.     Is reserved
7.     Is helpful and unselfish with others
8.     Can be somewhat careless
9.     Is relaxed, handles stress well. 
10.  Is curious about many different things
11.   Is full of energy
12.   Starts quarrels with others
13.   Is a reliable worker
14.   Can be tense
15.   Is ingenious, a deep thinker
16.   Generates a lot of enthusiasm
17.   Has a forgiving nature
18.   Tends to be disorganized
19.   Worries a lot
20.   Has an active imagination
21.   Tends to be quiet
22.   Is generally trusting
23.   Tends to be lazy
24.   Is emotionally stable, not easily upset
25.   Is inventive
26.   Has an assertive personality
27.   Can be cold and aloof
28.   Perseveres until the task is finished
29.   Can be moody
30.   Values artistic, aesthetic experiences
31.   Is sometimes shy, inhibited
32.   Is considerate and kind to almost everyone
33.   Does things efficiently
34.   Remains calm in tense situations
35.   Prefers work that is routine
36.   Is outgoing, sociable
37.   Is sometimes rude to others
38.   Makes plans and follows through with them
39.   Gets nervous easily
40.   Likes to reflect, play with ideas
41.   Has few artistic interests
42.   Likes to cooperate with others
43.   Is easily distracted
44.   Is sophisticated in art, music, or literature

## Big Five Inventory (Dutch)

De volgende stellingen hebben betrekking op uw opvatting over uzelf in verschillende situaties. Het is aan u om aan te geven in hoeverre u het eens bent met elke stelling, waarbij u gebruik maakt van een schaal waarop 1 helemaal oneens betekent, 5 helemaal eens betekent, en 2, 3 en 4 zijn beoordelingen daartussenin.

Ik zie mezelf als iemand die...

1. Spraakzaam is.
2. Geneigd is kritiek te hebben op anderen.
3. Grondig te werk gaat.
4. Somber is.
5. Origineel is, met nieuwe ideeën komt.
6. Terughoudend is.
7. Behulpzaam en onzelfzuchtig ten opzichte van anderen is.
8. Een beetje nonchalant kan zijn.
9. Ontspannen is, goed met stress kan omgaan.
10. Benieuwd is naar veel verschillende dingen.
11. Vol energie is.
12. Snel ruzie maakt.
13. Een werker is waar men van op aan kan.
14. Gespannen kan zijn.
15. Scherpzinnig, een denker is.
16. Veel enthousiasme opwekt.
17. Vergevingsgezind is.
18. Doorgaans geneigd is tot slordigheid.
19. Zich veel zorgen maakt.
20. Een levendige fantasie heeft.
21. Doorgaans stil is.
22. Mensen over het algemeen vertrouwt.
23. Geneigd is lui te zijn.
24. Emotioneel stabiel is, niet gemakkelijk overstuur raakt.
25. Vindingrijk is.
26. Voor zichzelf opkomt.
27. Koud en afstandelijk kan zijn.
28. Volhoudt tot de taak af is.
29. Humeurig kan zijn.
30. Waarde hecht aan kunstzinnige ervaringen.
31. Soms verlegen, geremd is.
32. Attent en aardig is voor bijna iedereen.
33. Dingen efficiënt doet.
34. Kalm blijft in gespannen situaties.
35. Een voorkeur heeft voor werk dat routine is.
36. Hartelijk, een gezelschapsmens is.
37. Soms grof tegen anderen is.
38. Plannen maakt en deze doorzet.
39. Gemakkelijk zenuwachtig wordt.
40. Graag nadenkt, met ideeën speelt.
41. Weinig interesse voor kunst heeft.
42. Graag samenwerkt met anderen.
43. Gemakkelijk afgeleid is.
44. Het fijne weet van kunst, muziek, of literatuur.

## Demographics (English)

What is your Gender?

1. Male
2. Female
3. Other
4. Prefer not to say

What age are you? ____

## Demographics (Dutch)

What is je geslacht?

1. Man
2. Vrouw
3. Anders
4. Zeg ik liever niet

Wat is jouw leeftijd? _____

# Study 2

On the next several pages, you will be asked to think of a person that you know personally who fits certain criteria. For each person, you will then be asked to rate their experiences and preferences based on your general impression of this person.

Please think of a DIFFERENT person for each section of the task (that is, do not repeat the same person twice). Your answers are anonymous. We will not ask your name, nor the names of the people that you think of (though we will ask you to type in their initials to show that you did think of a particular person). We will not be able to identify you or the people you think of, so please answer all questions as honestly as possible. Thank you.

We will ask you to think of a total of three different people.

- Please think of a family member.

Once you have thought of someone who fits this description, visualize them carefully

in your mind. Imagine how that person thinks, feels, and acts.

When you have a clear image of what this person is like, type their initials in the box below ____

## Peer ratings: Preference for solitude

Now please indicate how much each statement describes that same person [initials], a family member of yours.

| 1 | 2 | 3 | 4 | 5 | 6 | 7 |
| --- | --- | --- | --- | --- | --- | --- |
| Not at all |  |  |  |  |  | Very much |

This person does not like being alone.

Being apart from other people for long periods of time does not bother this person.

This person enjoys the pleasure of solitude.

This person sees himself/herself as a loner.

In this person's opinion, the perfect weekend is spent alone.

This person prefers spending Friday night alone rather than being with others.

In this person's opinion, the perfect vacation would be with lots of people around.

This person’s dream vacation is to be alone with no connections to others.

With free time, this person prefers to be with others.

This person thinks clearer when alone.

Solitude helps this person center himself/herself.

This person does not understand people who choose to be alone.

This person needs time each day alone to collect his/her thoughts.

Spending time alone enhances his/her day.

This person needs time alone each day.

For this person, being with others for extended amounts of time becomes unbearable.

## Peer ratings: Ostracism experiences

Below are several statements that can be used to describe people. Please rate how well each statement describes [initials], a family member of yours. If you don't know exactly, just use your best guess.

| 1  Hardly ever | 2 | 3 | 4 Sometimes | 5 | 6 | 7  Almost always |
| --- | --- | --- | --- | --- | --- | --- |

In general, others leave this person out of their group.

In general, others keep this person out-of-the-loop on information.

In general, others treat this person as if he/she was invisible.

In general, others give this person the cold shoulder treatment.

In general, others physically turn their backs to this person when in his/her presence.

In general, others treat this person as if he or she is in solitary confinement.

In general, others do not look at this person when he/she is in their presence.

In general, others ignore this person during their conversation.

## Peer ratings: The need to belong

| 1 | 2 | 3 | 4 | 5 | 6 | 7 |
| --- | --- | --- | --- | --- | --- | --- |
| Not at all |  |  |  |  |  | Very much |

This person has a strong need to belong.

- Please think of a friend who is of the same gender as you.
  - [same measures above]
- Please think of a person you have known for at least 4 months. You know this person moderately well but you would NOT consider his/her as a friend or a romantic partner.
  - [same measures above]

Now, for each of the following statements, please consider your personal feelings.

Determine how often, in general, the following experiences happen to you. Just give your gut response.

## Self ratings: Preference for solitude

To what extent does each of the following statements apply to you? Please respond using a 7-point scale:

| 1 | 2 | 3 | 4 | 5 | 6 | 7 |
| --- | --- | --- | --- | --- | --- | --- |
| Not at all |  |  |  |  |  | Very much |

I do not like being alone.

Being apart from other people for long periods of time does not bother me.

I enjoy the pleasure of solitude.

I see myself as a loner.

The perfect weekend is spent alone.

I prefer spending Friday night alone rather than being with others.

The perfect vacation would be with lots of people around.

My dream vacation is to be alone with no connections to others.

With free time, I prefer to be with others.

I feel I can think clearer when alone.

Solitude helps me center myself.

I do not understand people who choose to be alone.

I need time each day alone to collect my thoughts.

Spending time alone enhances my day.

I need time alone each day.

Being with others for extended amounts of time becomes unbearable.

## Self ratings: Ostracism experiences

For each of the following statements, please consider your personal feelings. Determine how often, in general, the following experiences happen to you. Just give your gut response. Use the sale provided:

| 1 | 2 | 3 | 4 | 5 | 6 | 7 |
| --- | --- | --- | --- | --- | --- | --- |
| Hardly ever |  |  | Sometimes |  |  | Almost always |

In general, others leave me out of their group.

In general, others keep me out-of-the-loop on information.

In general, others treat me as if I am invisible.

In general, others give me the cold shoulder treatment.

In general, others physically turn their backs to me when in my presence.

In general, others treat me as if I’m in solitary confinement.

In general, others do not look at me when I’m in their presence.

In general, others ignore me during their conversation.

## Self ratings: The need to belong

| 1 | 2 | 3 | 4 | 5 | 6 | 7 |
| --- | --- | --- | --- | --- | --- | --- |
| Not at all |  |  |  |  |  | Very much |

I have a strong need to belong.

## Demographics

Sex: Male Female

Age: dropdown list: 1-100

# Study 3

Suppose you are going to meet with a person for the first time. You had no prior knowledge of this person. Before you meet with this person, you got a profile of this person. You are going to read this profile on the next page. Please form an impression as you read it.

## Manipulation

Please read the profile carefully, because you will be asked to evaluate this person based on the information provided.

Condition: low preference for solitude

*I am a student at [the name of the university] and this is my third year in college. My favorite season of the year is Fall because it is nice outside. I am the kind of person who doesn’t prefer being alone. I try to attend most social events I'm invited to. I don’t really need “me time” and being apart from other people for long periods of time bothers me. With free time, I usually want to have friends around, watching movies, enjoying good food or just hanging out. My dream vacation is to be with people and make social connections.*

Make sure you form an impression before you proceed. You will be asked to evaluate this person based on the impression you formed.

Condition: high preference for solitude

*I am a student at [the name of the university] and this is my third year in college. My favorite season of the year is Fall because it is nice outside. I am the kind of person who prefers being alone. I struggle to attend social events I'm invited to. I need “me time” each day and being apart from other people for long periods of time does not bother me. With free time, I usually want to be alone, watching movies, enjoying good food or just chilling out. My dream vacation is to be alone with no connections to others.*

Make sure you form an impression before you proceed. You will be asked to evaluate this person based on the impression you formed.

## The Big Five

Here are a number of characteristics that may or may not apply to the person you just read about. For example, do you agree that this person is someone who likes to spend time with others? Please choose the number next to each statement to indicate the extent to which you agree or disagree with that statement.

This person is someone who...

| 1 | 2 | 3 | 4 | 5 |
| --- | --- | --- | --- | --- |
| Disagree  Strongly | Disagree  a little | Neither agree  nor disagree | Agree  a little | Agree  strongly |

1.     Is talkative
2.     Tends to find fault with others
3.     Does a thorough job
4.     Is depressed, blue
5.     Is original, comes up with new ideas
6.     Is reserved
7.     Is helpful and unselfish with others
8.     Can be somewhat careless
9.     Is relaxed, handles stress well. 
10.  Is curious about many different things
11.   Is full of energy
12.   Starts quarrels with others
13.   Is a reliable worker
14.   Can be tense
15.   Is ingenious, a deep thinker
16.   Generates a lot of enthusiasm
17.   Has a forgiving nature
18.   Tends to be disorganized
19.   Worries a lot
20.   Has an active imagination
21.   Tends to be quiet
22.   Is generally trusting
23.   Tends to be lazy
24.   Is emotionally stable, not easily upset
25.   Is inventive
26.   Has an assertive personality
27.   Can be cold and aloof
28.   Perseveres until the task is finished
29.   Can be moody
30.   Values artistic, aesthetic experiences
31.   Is sometimes shy, inhibited
32.   Is considerate and kind to almost everyone
33.   Does things efficiently
34.   Remains calm in tense situations
35.   Prefers work that is routine
36.   Is outgoing, sociable
37.   Is sometimes rude to others
38.   Makes plans and follows through with them
39.   Gets nervous easily
40.   Likes to reflect, play with ideas
41.   Has few artistic interests
42.   Likes to cooperate with others
43.   Is easily distracted
44.   Is sophisticated in art, music, or literature

## Person perception

These traits may or may not apply to the person you read about. Please rate on the following scale: to what extent would you consider these traits apply to the person you read about?

| 1 | 2 | 3 | 4 | 5 | 6 | 7 |
| --- | --- | --- | --- | --- | --- | --- |
| Not at all |  |  |  |  |  | Very much so |

kind, warm, friendly, competent, intelligent, smart

## The need to belong

Indicate the degree to which you agree or disagree with the statement by choosing a number using the scale below:

| 1 | 2 | 3 | 4 | 5 |
| --- | --- | --- | --- | --- |
| Strongly  Disagree | Moderately disagree | Neither agree  nor disagree | Moderately agree | Strongly agree |

This person has a strong need to belong.

## Reactions to belonging events

For each of the statements below, indicate the degree to which you agree or disagree with the statement by choosing a number using the scale below:

| 1 | 2 | 3 | 4 | 5 |
| --- | --- | --- | --- | --- |
| Strongly  Disagree | Moderately disagree | Neither agree  nor disagree | Moderately agree | Strongly agree |

Being included in a group conversation would make this person feel very good.

Being part of a group activity would make this person very happy.

Being ignored would hurt this person's feelings very much.

This person would be bothered a great deal when they are not included in other people's plans.

This person would be easily affected when they feel that others do not accept them.

If other people don't seem to accept this person, this person wouldn't be bothered for long.

If this person were ignored by others, they would be able to recover from it easily.

If other people excluded this person from a group activity, this person would get over it quickly.

## Ostracism intentions

Based on your impressions of this person, answer following questions about your feelings towards this person:

| 1 | 2 | 3 | 4 | 5 |
| --- | --- | --- | --- | --- |
| Not at all |  |  |  | Very much |

I would want to be friends with this person.

I would have little interest in interacting with this person.

I would be interested in forming a connection with this person.

I would leave this person out of my group.

I would invite this person to events.

I would ignore this person during conversations.

I would give this person little attention in a group.

I would want to meet with this person.

## Manipulation check

Indicate the degree to which you agree or disagree with the statement by choosing a number using the scale below:

| 1 | 2 | 3 | 4 | 5 |
| --- | --- | --- | --- | --- |
| Strongly  Disagree | Moderately disagree | Neither agree  nor disagree | Moderately agree | Strongly agree |

This person prefers solitude.

## Demographics

What is your sex? Male Female

What is your age? ____

# Study 4

Earlier this semester we conducted a study where we had participants answer one questionnaire. This questionnaire has 16 statements and participants were asked to indicate whether each statement applied to them on a 7-point scale (1= not at all, 7 = very much). Now, we are interested in how people form an impression based on others’ responses to this questionnaire.

On the next page, we will provide a screen shot of a completed questionnaire from one of our participants. Please read through the completed questionnaire carefully and observe how this person rated each statement. Based on this person's responses, please form an impression.

Please read the completed questionnaire carefully, because you will be asked to evaluate this person based on the information provided.

## Manipulation

Here is a screen shot of the completed questionnaire by one of our participants. Please read carefully how this person rated each statement:


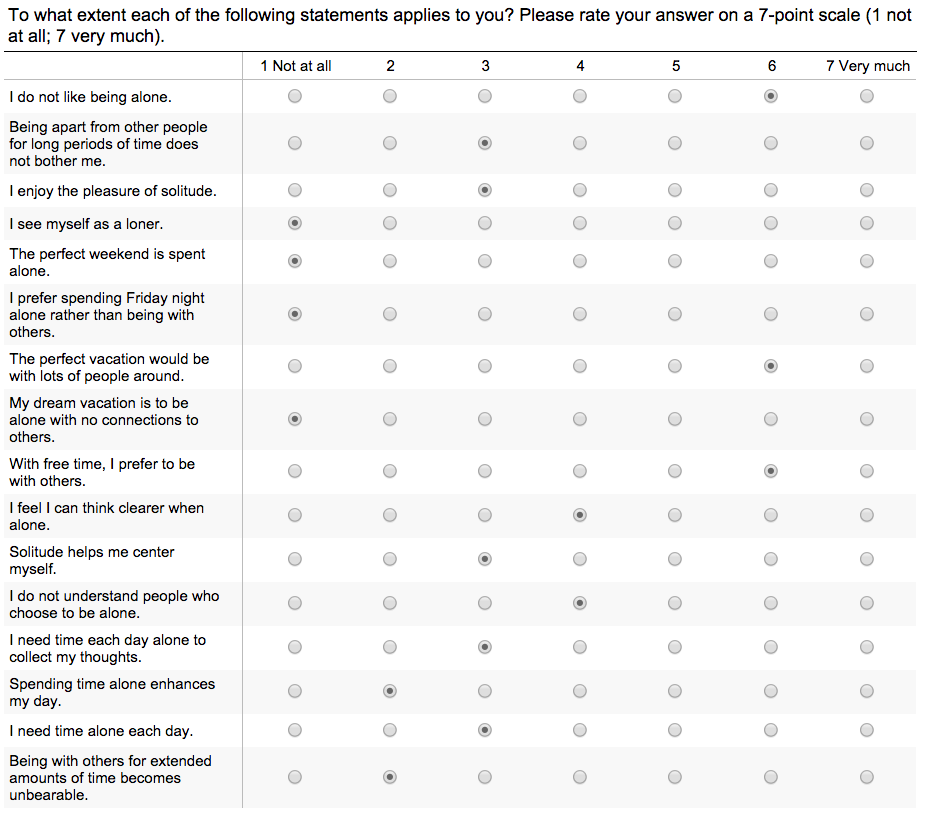
Condition: low preference for solitude

Condition: average preference for solitude


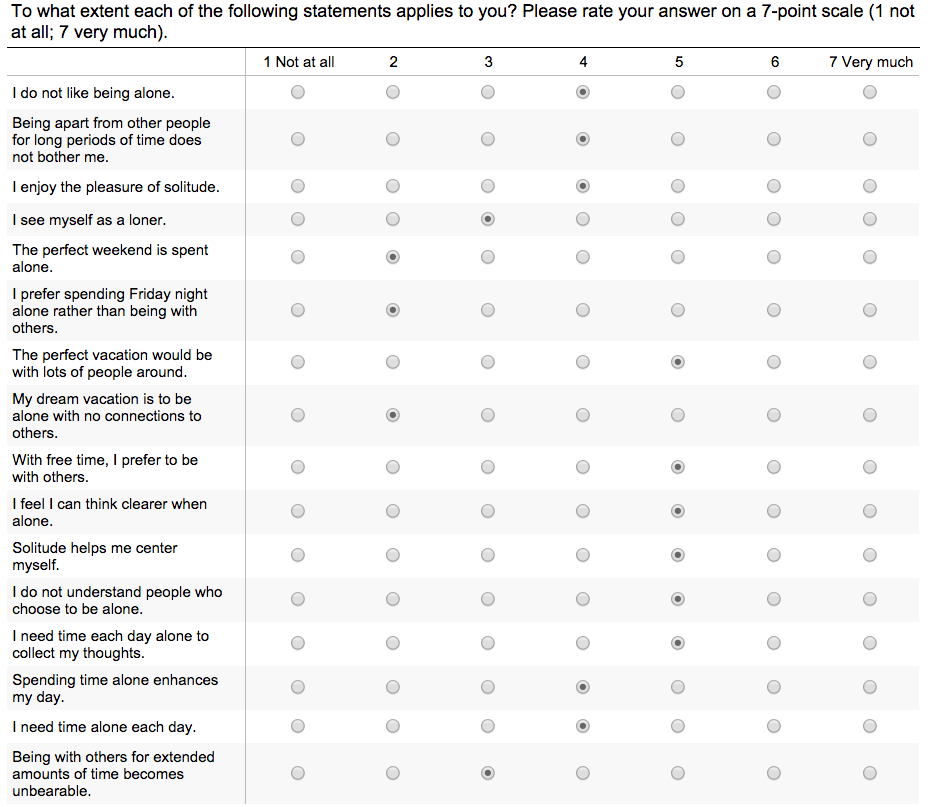


Condition: high preference for solitude


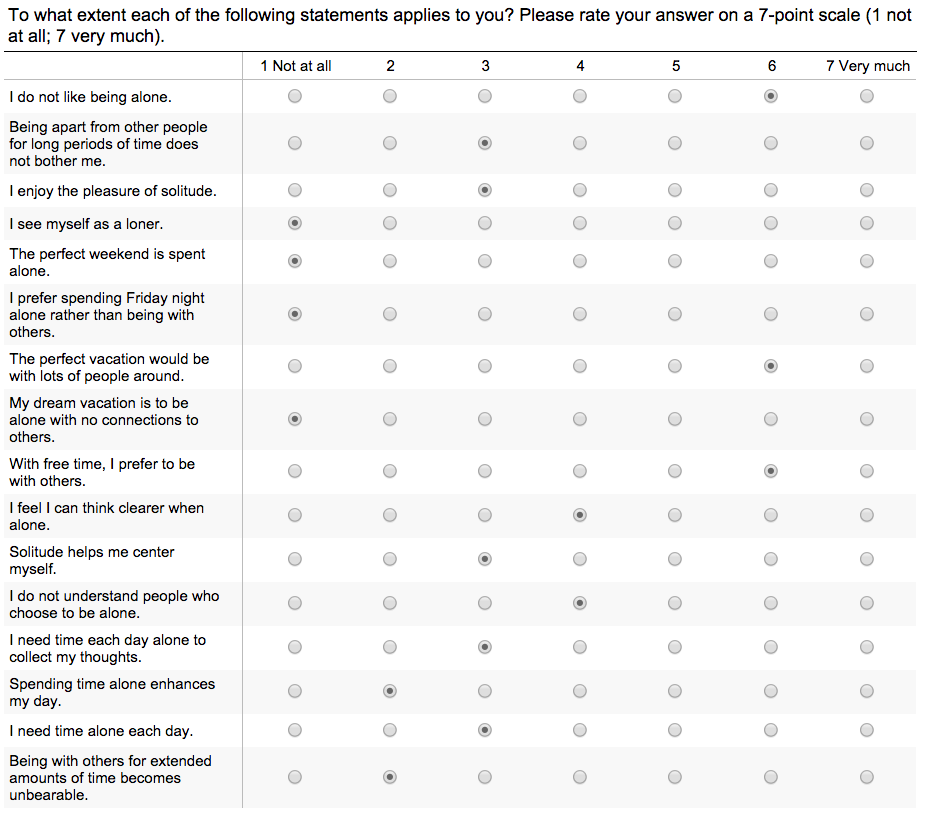


Make sure you form an impression before you proceed. You will be asked to evaluate this person based on the impression you formed.

## The Big Five

Here are a number of characteristics that may or may not apply to the person you just read about. For example, do you agree that this person is someone who likes to spend time with others? Please choose the number next to each statement to indicate the extent to which you agree or disagree with that statement.

This person is someone who...

| 1 | 2 | 3 | 4 | 5 |
| --- | --- | --- | --- | --- |
| Disagree  Strongly | Disagree  a little | Neither agree  nor disagree | Agree  a little | Agree  strongly |

1.     Is talkative
2.     Tends to find fault with others
3.     Does a thorough job
4.     Is depressed, blue
5.     Is original, comes up with new ideas
6.     Is reserved
7.     Is helpful and unselfish with others
8.     Can be somewhat careless
9.     Is relaxed, handles stress well. 
10.  Is curious about many different things
11.   Is full of energy
12.   Starts quarrels with others
13.   Is a reliable worker
14.   Can be tense
15.   Is ingenious, a deep thinker
16.   Generates a lot of enthusiasm
17.   Has a forgiving nature
18.   Tends to be disorganized
19.   Worries a lot
20.   Has an active imagination
21.   Tends to be quiet
22.   Is generally trusting
23.   Tends to be lazy
24.   Is emotionally stable, not easily upset
25.   Is inventive
26.   Has an assertive personality
27.   Can be cold and aloof
28.   Perseveres until the task is finished
29.   Can be moody
30.   Values artistic, aesthetic experiences
31.   Is sometimes shy, inhibited
32.   Is considerate and kind to almost everyone
33.   Does things efficiently
34.   Remains calm in tense situations
35.   Prefers work that is routine
36.   Is outgoing, sociable
37.   Is sometimes rude to others
38.   Makes plans and follows through with them
39.   Gets nervous easily
40.   Likes to reflect, play with ideas
41.   Has few artistic interests
42.   Likes to cooperate with others
43.   Is easily distracted
44.   Is sophisticated in art, music, or literature

## Person perception

These traits may or may not apply to the person you read about. Please rate on the following scale: to what extent would you consider these traits apply to the person you read about?

| 1 | 2 | 3 | 4 | 5 | 6 | 7 |
| --- | --- | --- | --- | --- | --- | --- |
| Not at all |  |  |  |  |  | Very much so |

kind, warm, friendly, competent, intelligent, smart

## The need to belong

Indicate the degree to which you agree or disagree with the statement by choosing a number using the scale below:

| 1 | 2 | 3 | 4 | 5 |
| --- | --- | --- | --- | --- |
| Strongly  Disagree | Moderately disagree | Neither agree  nor disagree | Moderately agree | Strongly agree |

This person has a strong need to belong.

## Reactions to belonging events

For each of the statements below, indicate the degree to which you agree or disagree with the statement by choosing a number using the scale below:

| 1 | 2 | 3 | 4 | 5 |
| --- | --- | --- | --- | --- |
| Strongly  Disagree | Moderately disagree | Neither agree  nor disagree | Moderately agree | Strongly agree |

Being included in a group would make this person feel very good.

Being ignored would hurt this person's feelings very much.

If this person were ignored by others, they would be able to recover from it easily.

If other people excluded this person from a group activity, this person would get over it quickly.

## Ostracism Intentions

Based on your impressions of this person, answer following questions about your feelings towards this person:

| 1 | 2 | 3 | 4 | 5 |
| --- | --- | --- | --- | --- |
| Not at all |  |  |  | Very much |

I would want to be friends with this person.

I would have little interest in interacting with this person.

I would be interested in forming a connection with this person.

I would leave this person out of my group.

I would invite this person to events.

I would ignore this person during conversations.

I would give this person little attention in a group.

I would want to meet with this person.

## Manipulation check

Indicate the degree to which you agree or disagree with the statement by choosing a number using the scale below:

| 1 | 2 | 3 | 4 | 5 |
| --- | --- | --- | --- | --- |
| Strongly  Disagree | Moderately disagree | Neither agree  nor disagree | Moderately agree | Strongly agree |

This person prefers solitude.

## Demographics

What is your sex? Male Female

What is your age? ____

# Study 5

Earlier this semester we conducted a study where we had participants answer one questionnaire. This questionnaire has 16 statements and participants were asked to indicate whether each statement applied to them on a 7-point scale (1= not at all, 7 = very much). Now, we are interested in how people form an impression based on others' responses to this questionnaire.

On the next page, we will provide a screen shot of a completed questionnaire from one of our participants. Please read through the completed questionnaire carefully and observe how this person rated each statement. Based on this person's responses, please form an impression.

Please read the completed questionnaire carefully, because you will be asked to evaluate this person based on the information provided.

## Manipulation

Here is a screen shot of the completed questionnaire by one of our participants. Please read carefully how this person rated each statement. There are 16 statements in total.

Condition: low preference for solitude


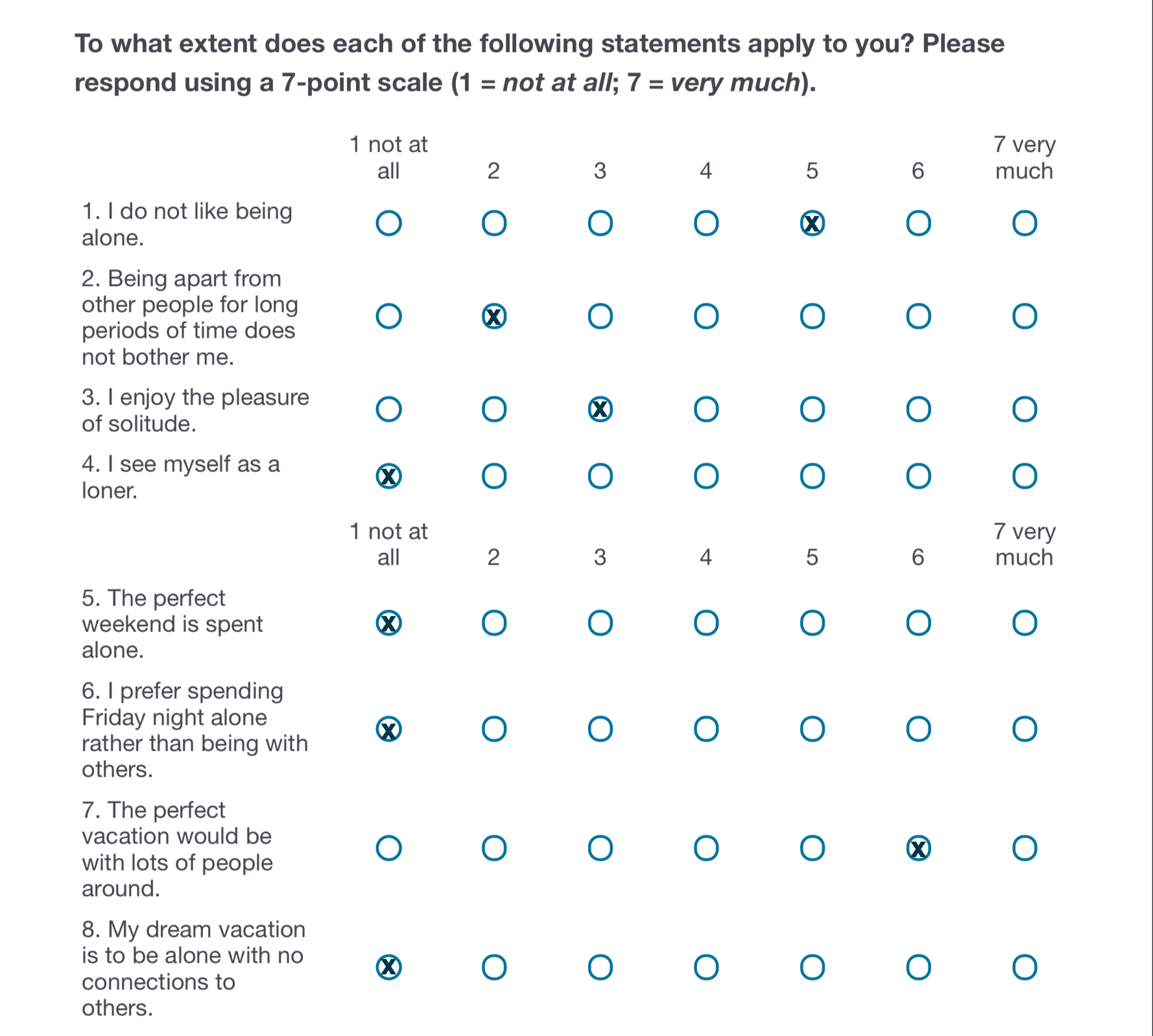

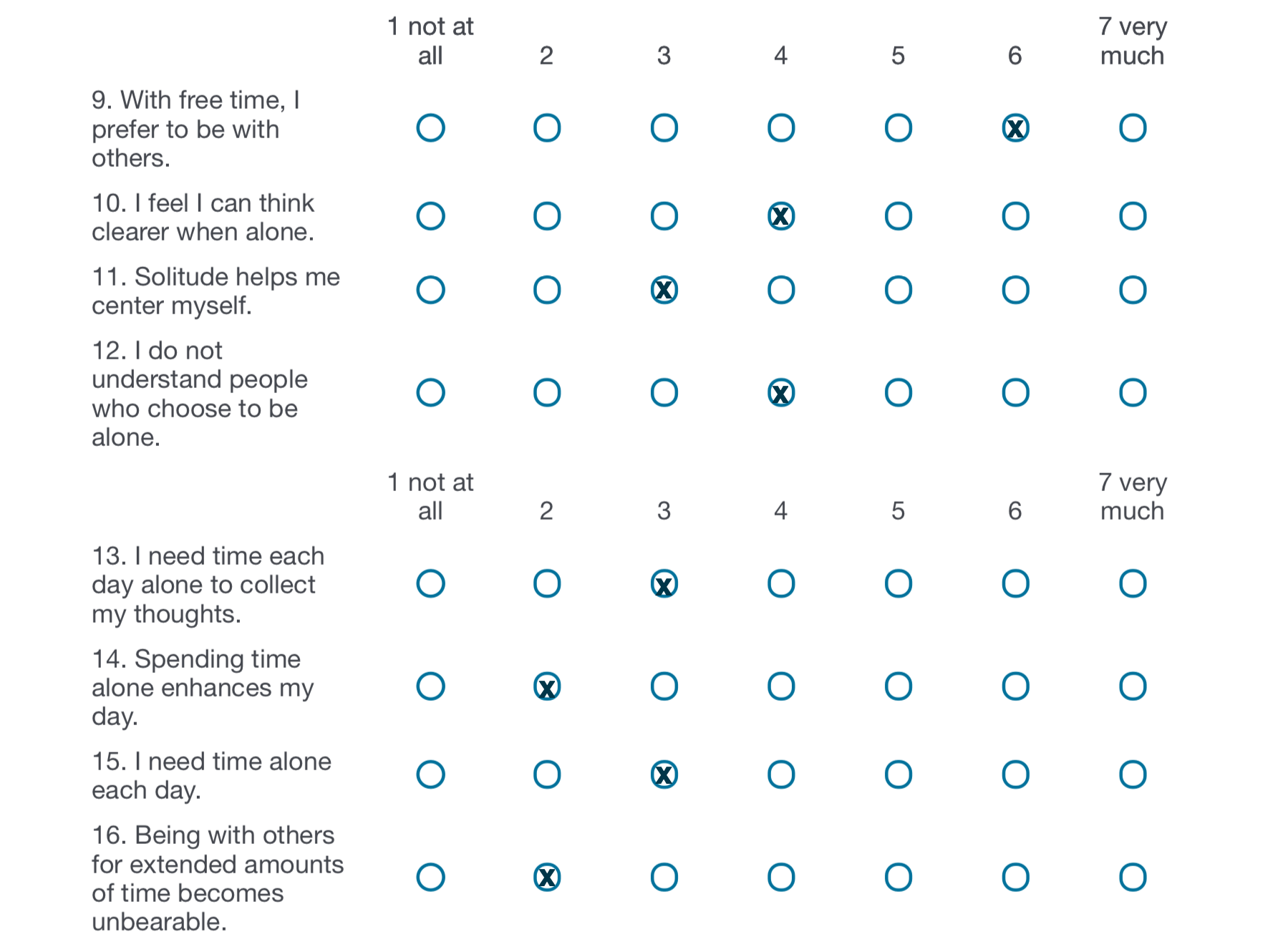


Condition: high preference for solitude


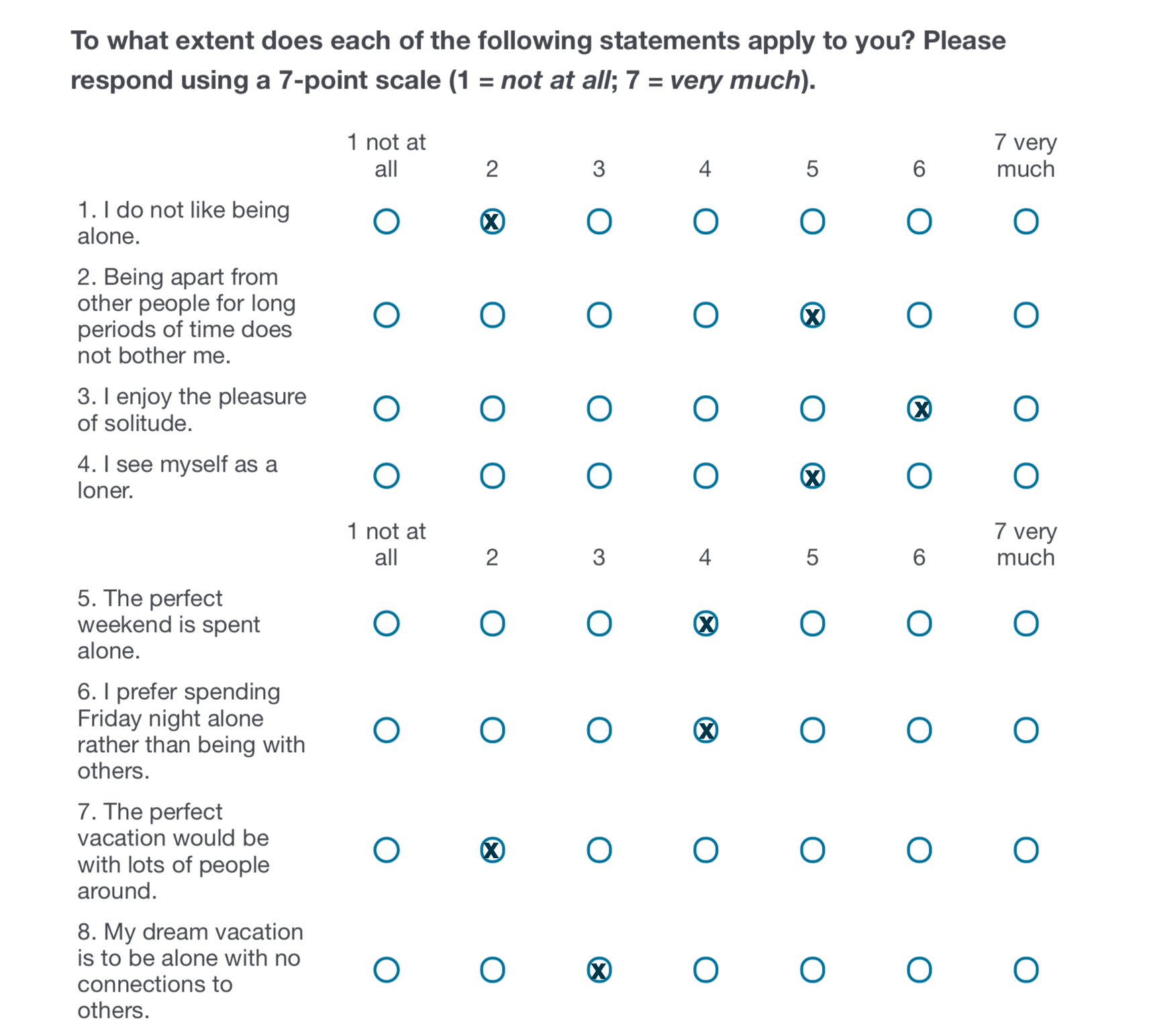

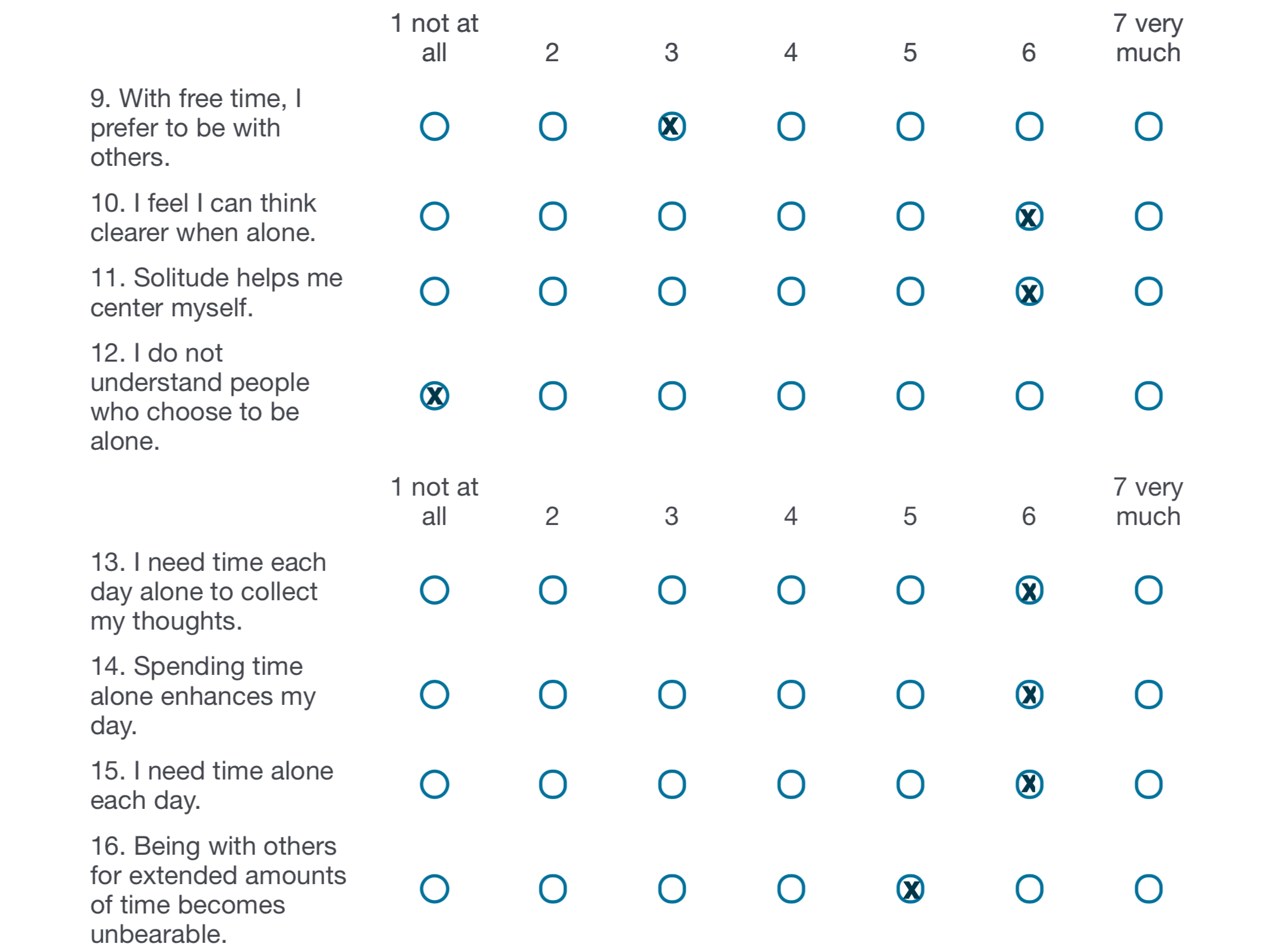


Make sure you form an impression before you proceed. You will be asked to evaluate this person based on the impression you formed.

I confirm I have formed an impression:

## Self-interested concerns

Based on your impressions of this person, answer following questions about this person:

| 1 | 2 | 3 | 4 | 5 |
| --- | --- | --- | --- | --- |
| Not at all |  |  |  | Very much |

I would probably not have a good time hanging out with this person at social events.

I don’t think I would enjoy interacting with this person.

I would not enjoy being around this person.

## Other-regarding concerns

Based on your impressions of this person, answer following questions about this person:

| 1 | 2 | 3 | 4 | 5 |
| --- | --- | --- | --- | --- |
| Not at all |  |  |  | Very much |

This person would probably not have a good time at social events.

I don't think this person would enjoy social interactions.

This person would not enjoy being around people.

## Ostracism intentions

Based on your impressions of this person, answer following questions about what you might consider doing:

| 1 | 2 | 3 | 4 | 5 |
| --- | --- | --- | --- | --- |
| Not at all |  |  |  | Very much |

I might find myself ignoring this person.

I might find myself giving this person little attention in a group.

I might consider leaving this person out of my group.

I would invite this person to events.

## Person perception

These traits may or may not apply to the person you read about. Please rate on the following scale: to what extent would you consider these traits apply to the person you read about?

| 1 | 2 | 3 | 4 | 5 | 6 | 7 |
| --- | --- | --- | --- | --- | --- | --- |
| Not at all |  |  |  |  |  | Very much so |

kind, warm, friendly, competent, intelligent, smart

## The Big Five

Here are a number of characteristics that may or may not apply to the person you just read about. For example, do you agree that this person is someone who likes to spend time with others? Please choose the number next to each statement to indicate the extent to which you agree or disagree with that statement.

This person is someone who...

| 1 | 2 | 3 | 4 | 5 |
| --- | --- | --- | --- | --- |
| Disagree  Strongly | Disagree  a little | Neither agree  nor disagree | Agree  a little | Agree  strongly |

1.     Is talkative
2.     Tends to find fault with others
3.     Does a thorough job
4.     Is depressed, blue
5.     Is original, comes up with new ideas
6.     Is reserved
7.     Is helpful and unselfish with others
8.     Can be somewhat careless
9.     Is relaxed, handles stress well. 
10.  Is curious about many different things
11.   Is full of energy
12.   Starts quarrels with others
13.   Is a reliable worker
14.   Can be tense
15.   Is ingenious, a deep thinker
16.   Generates a lot of enthusiasm
17.   Has a forgiving nature
18.   Tends to be disorganized
19.   Worries a lot
20.   Has an active imagination
21.   Tends to be quiet
22.   Is generally trusting
23.   Tends to be lazy
24.   Is emotionally stable, not easily upset
25.   Is inventive
26.   Has an assertive personality
27.   Can be cold and aloof
28.   Perseveres until the task is finished
29.   Can be moody
30.   Values artistic, aesthetic experiences
31.   Is sometimes shy, inhibited
32.   Is considerate and kind to almost everyone
33.   Does things efficiently
34.   Remains calm in tense situations
35.   Prefers work that is routine
36.   Is outgoing, sociable
37.   Is sometimes rude to others
38.   Makes plans and follows through with them
39.   Gets nervous easily
40.   Likes to reflect, play with ideas
41.   Has few artistic interests
42.   Likes to cooperate with others
43.   Is easily distracted
44.   Is sophisticated in art, music, or literature

## Demographics

What is your gender? Male Female Other

What is your age? dropdown list: 18-80
