# Supplementary material for: Leaving the Loners Alone: Dispositional Preference for Solitude Evokes Ostracism
Source: Pers Soc Psychol Bull. 2020 Nov 2;47(8):1294–308. doi: 10.1177/0146167220968612 (PMC8258721; doi:10.1177/0146167220968612)

**Supplementary Materials**

[Perceived belonging need in Study 2 1](#_Toc50146772)

[Manipulation materials in Study 3 1](#_Toc50146773)

[Manipulation materials in Studies 4 and 5 2](#_Toc50146774)

[Perceptions of targets: additional results 4](#_Toc50146775)

[Exploratory analysis: observer (participant) gender (Studies 3-5) 5](#_Toc50146776)

# Perceived belonging need in Study 2

We measured participants’ ratings of the target’s belonging need (“This person has a strong need to belong”) on a 7-point scale (1 = not at all, 7 = very much). Using the same analytic approach as in our main analysis, we observed a negative association between participants’ ratings of preference for solitude and ratings of belonging need, *b* = -0.70, 95% *CI* = [-0.76, -0.63], *p* < .001. This finding converged with the experimental results on perceived belonging need from Studies 3-5. Together, these results show that individuals with higher preference for solitude are perceived to have weaker need to belong.

# Manipulation materials in Study 3

High preference for solitude: I am a student at [name of the university] and this is my third year in college. My favorite season of the year is Fall because it is nice outside. I am the kind of person who prefers being alone. I struggle to attend social events I'm invited to. I need “me time” each day and being apart from other people for long periods of time does not bother me. With free time, I usually want to be alone, watching movies, enjoying good food or just chilling out. My dream vacation is to be alone with no connections to others.

Low preference for solitude: I am a student at [the name of the university] and this is my third year in college. My favorite season of the year is Fall because it is nice outside. I am the kind of person who doesn’t prefer being alone. I try to attend most social events I'm invited to. I don’t really need “me time” and being apart from other people for long periods of time bothers me. With free time, I usually want to have friends around, watching movies, enjoying good food or just hanging out. My dream vacation is to be with people and make social connections.

# Manipulation materials in Studies 4 and 5

The preference for solitude measure was ostensibly filled in by a fellow student – the target (Study 4: preference for solitude: low, average, high; Study 5: low, high). The responses to each item are indicated in Supplementary Table 1. The images we used as manipulation materials are presented in Supplementary Figures 1 and 2.

**Supplementary Table 1**

Item scores (Studies 4 & 5).

|  | Study 4 targets | | | Study 5 targets | |
| --- | --- | --- | --- | --- | --- |
| Items | low | average | high | low | high |
| 1.     I do not like being alone. | 6 | 4 | 3 | 5 | 2 |
| 2.     Being apart from other people for long periods of time does not bother me. | 3 | 4 | 5 | 2 | 5 |
| 3.     I enjoy the pleasure of solitude. | 3 | 4 | 6 | 3 | 6 |
| 4.     I see myself as a loner. | 1 | 3 | 5 | 1 | 5 |
| 5.     The perfect weekend is spent alone. | 1 | 2 | 4 | 1 | 4 |
| 6.     I prefer spending Friday night alone rather than being with others. | 1 | 2 | 4 | 1 | 4 |
| 7.     The perfect vacation would be with lots of people around. | 6 | 5 | 3 | 6 | 2 |
| 8.     My dream vacation is to be alone with no connections to others. | 1 | 2 | 3 | 1 | 3 |
| 9.     With free time, I prefer to be with others. | 6 | 5 | 4 | 6 | 3 |
| 10.  I feel I can think clearer when alone. | 4 | 5 | 6 | 4 | 6 |
| 11.  Solitude helps me center myself. | 3 | 5 | 6 | 3 | 6 |
| 12.  I do not understand people who choose to be alone. | 4 | 3 | 2 | 4 | 1 |
| 13.  I need time each day alone to collect my thoughts. | 3 | 5 | 6 | 3 | 6 |
| 14.  Spending time alone enhances my day. | 2 | 4 | 6 | 2 | 6 |
| 15.  I need time alone each day. | 3 | 4 | 6 | 3 | 6 |
| 16.  Being with others for extended amounts of time becomes unbearable. | 2 | 3 | 5 | 2 | 5 |

**Supplementary Figure 1**


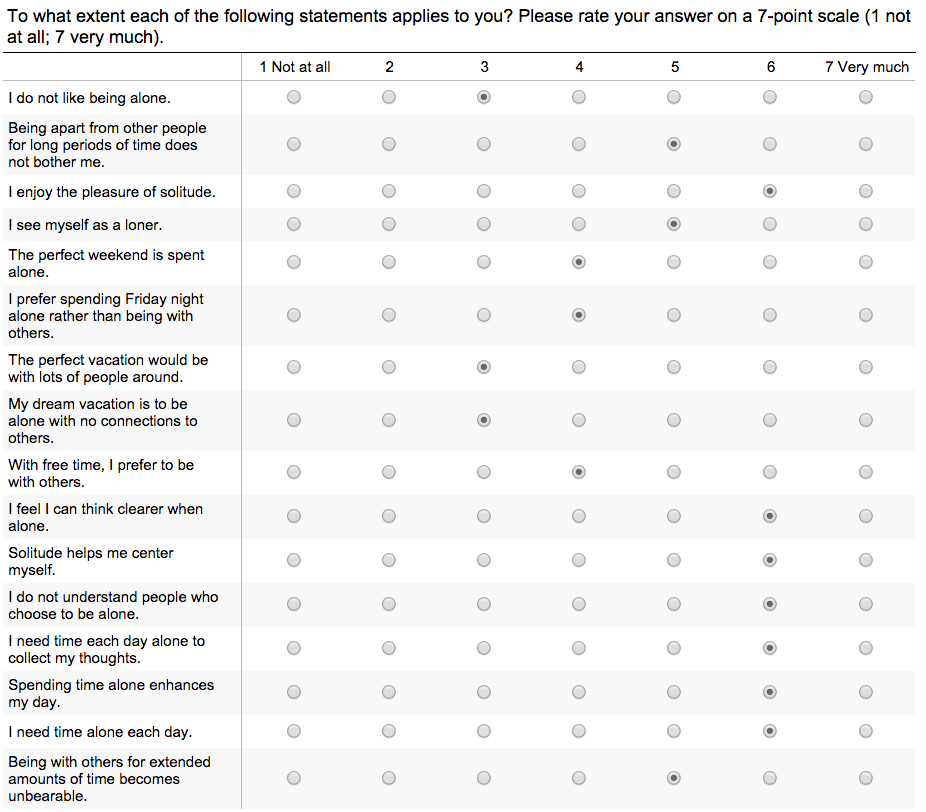

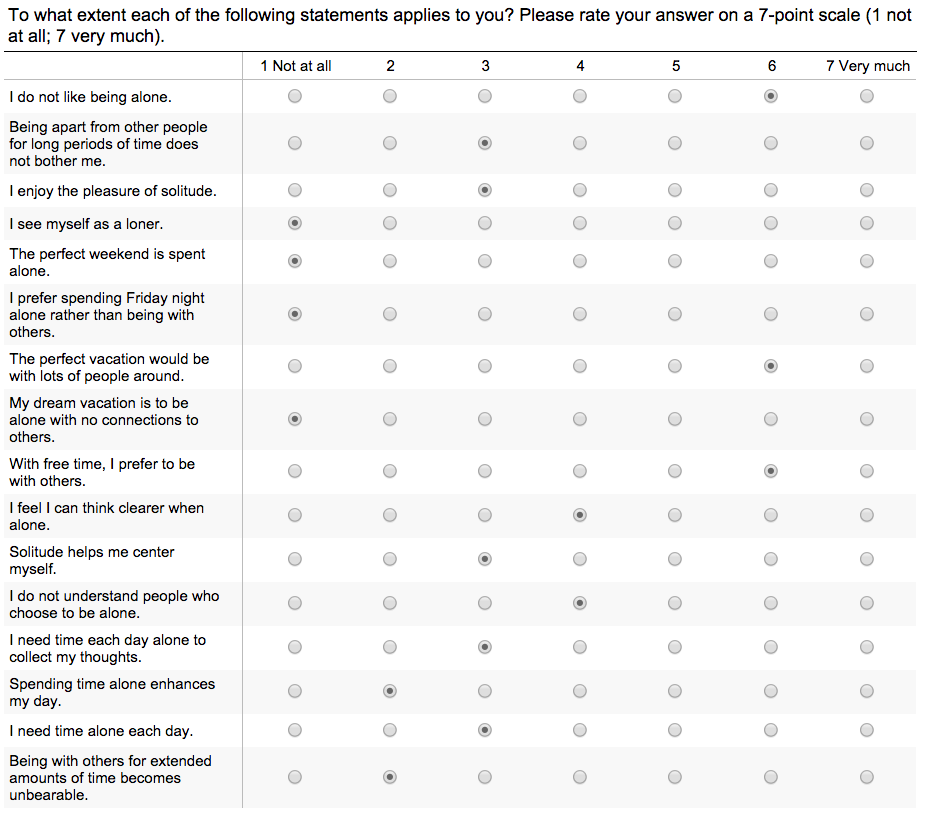

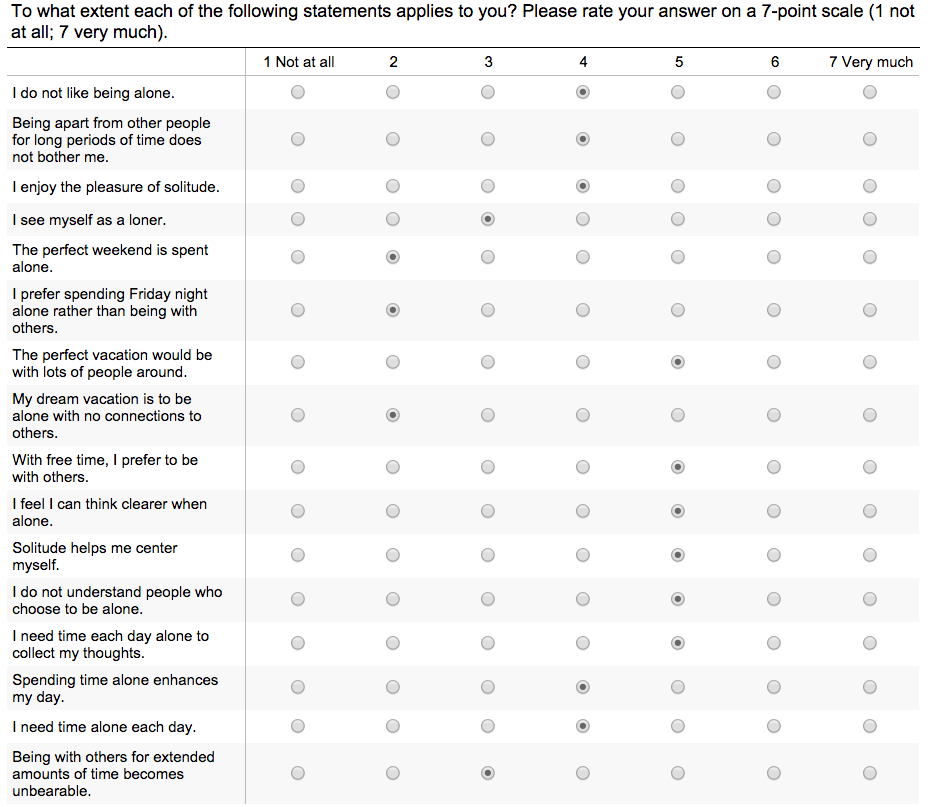
Manipulated preference for solitude in Study 4: Low (top), average (middle), and High (bottom).

**Supplementary Figure 2**

Manipulated preference for solitude in Study 5: Low (left) and High (right).


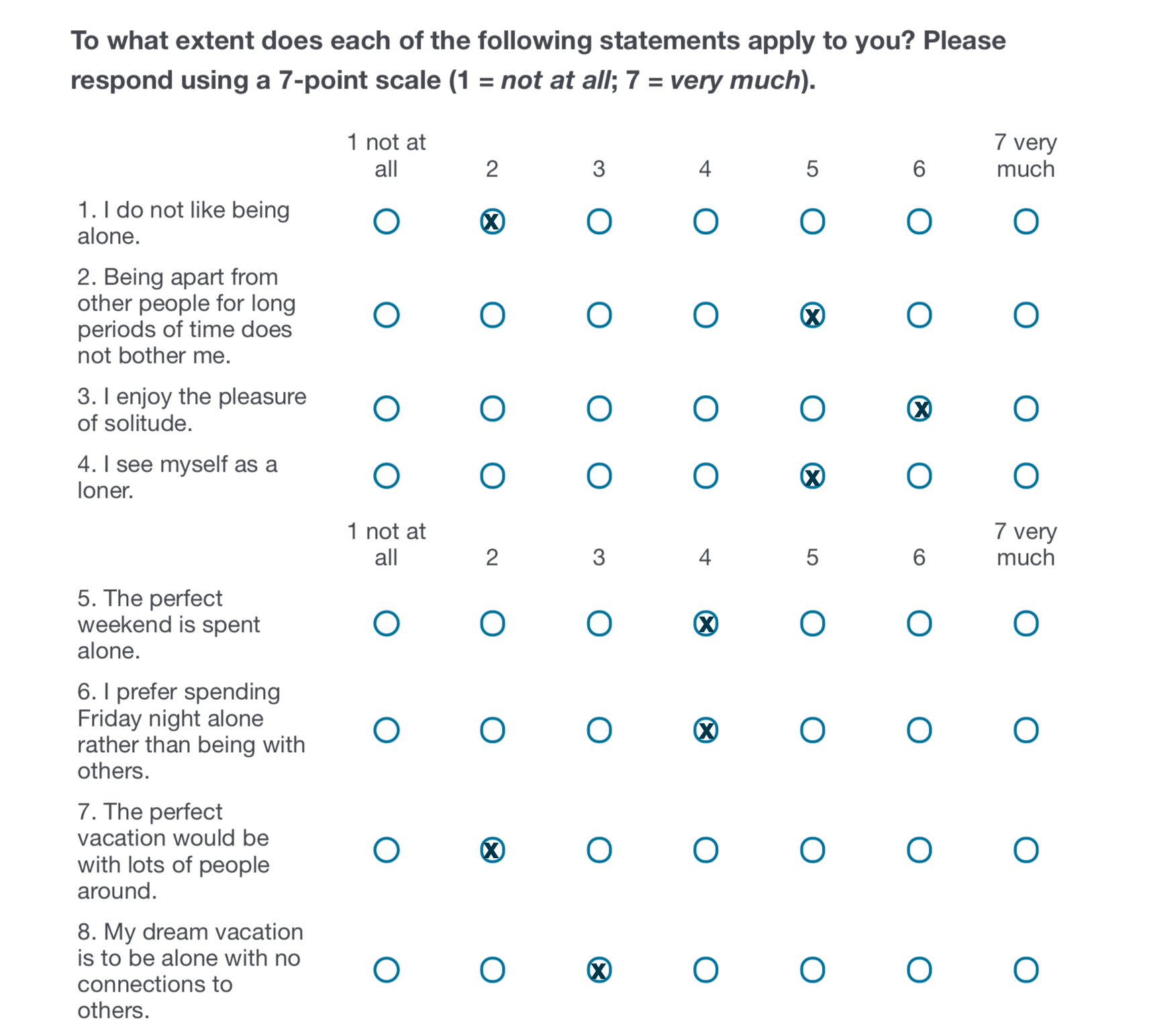

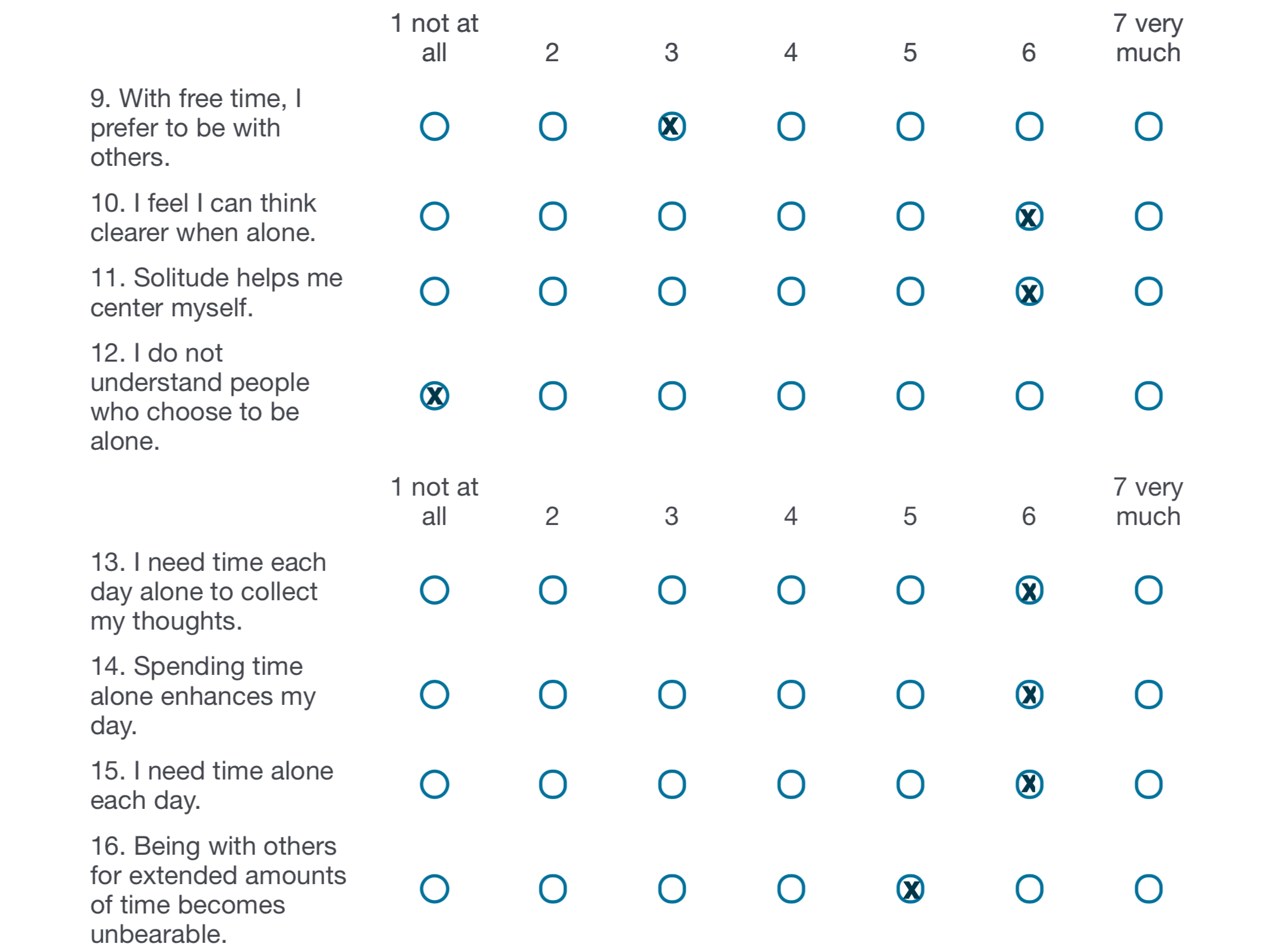

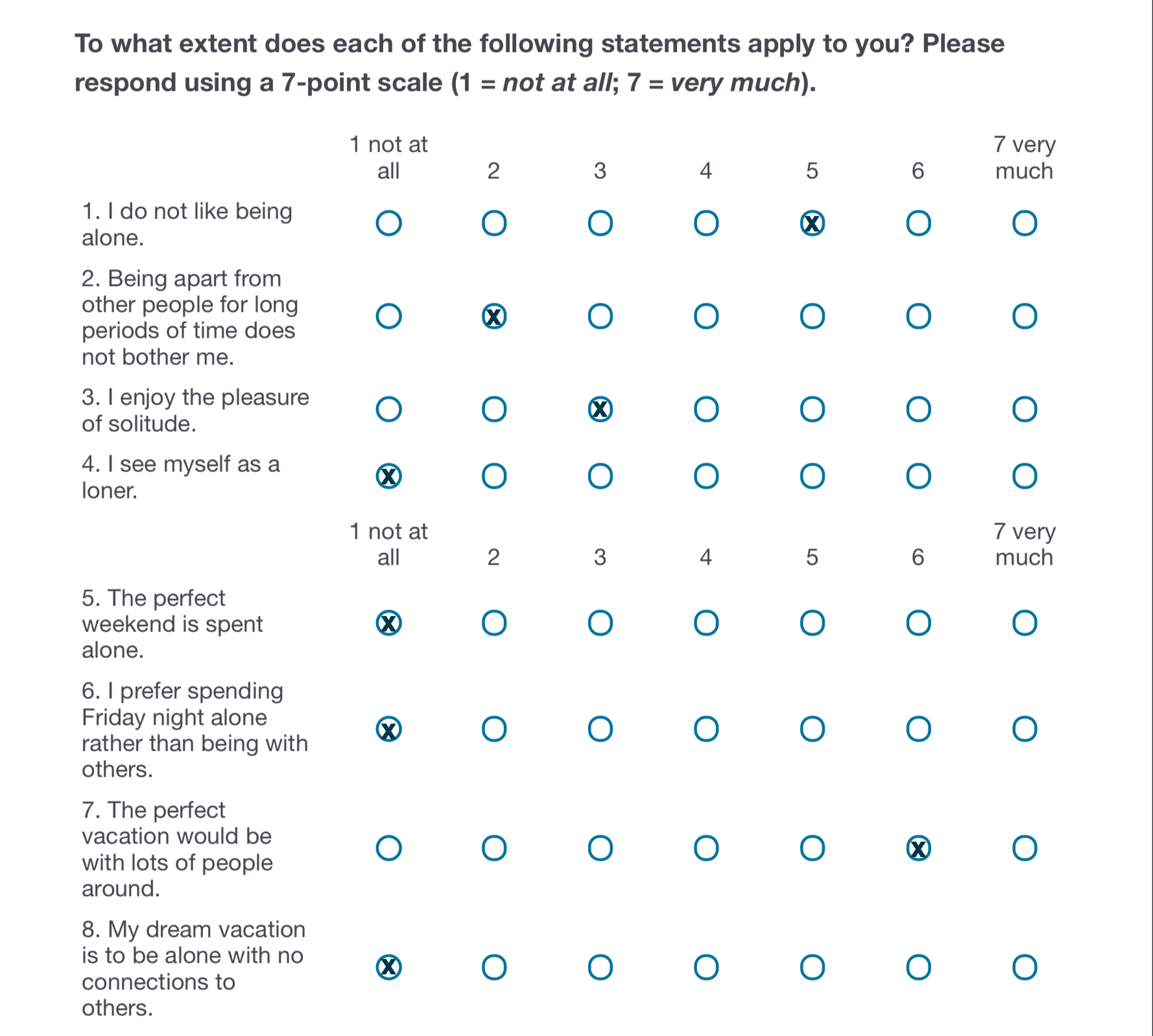

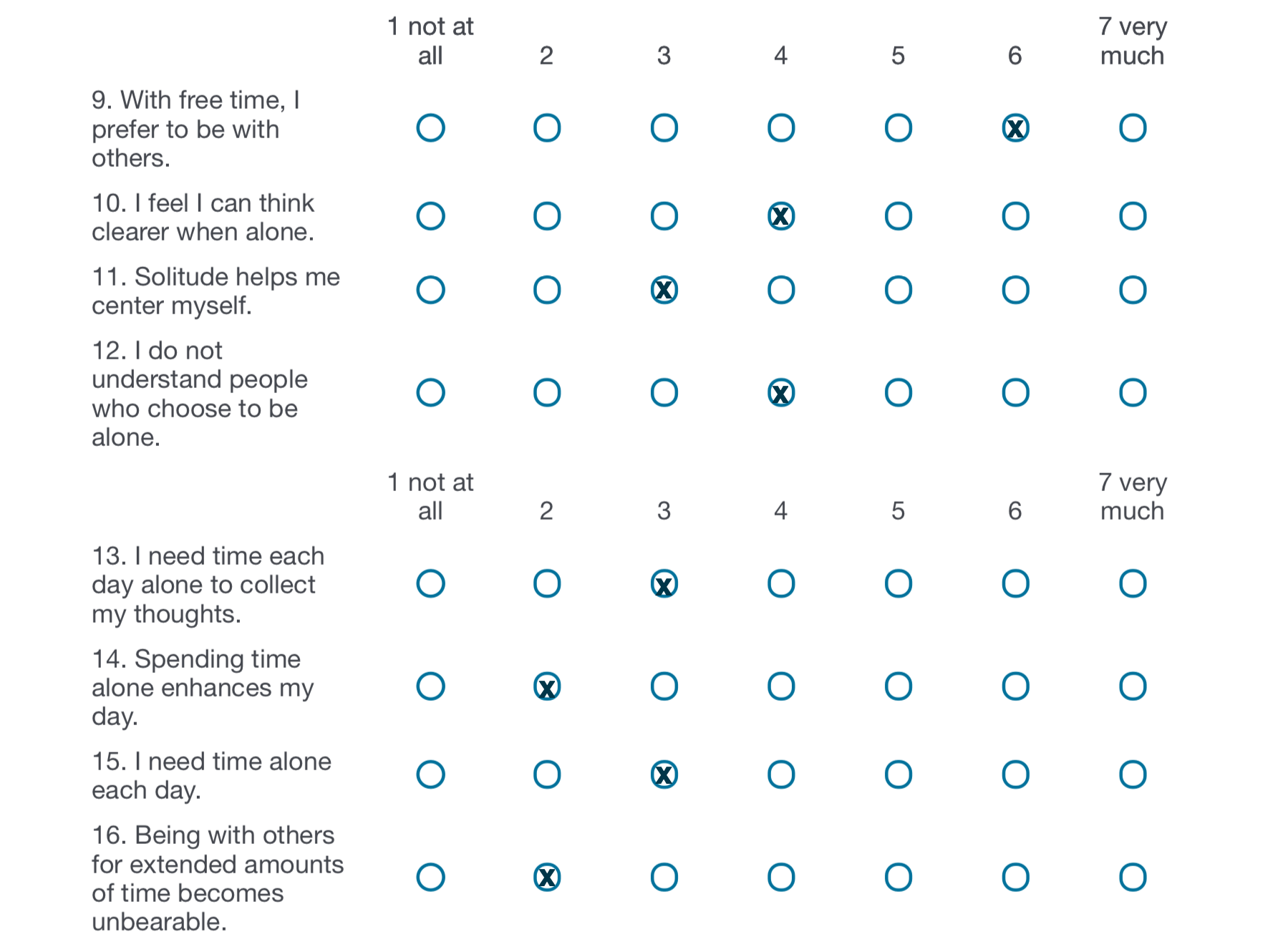


# Perceptions of targets: additional results

In addition to the effects of having a high (vs. low) preference for solitude we have reported in the main text, we also explored the effects of having an average (vs. low) preference for solitude, and the effects of having a high (vs. average) preference for solitude in Study 4. These additional effects are summarized in Supplementary Table 2.

**Supplementary Table 2**

Perceptions of targets (Study 4).

|  | Average (ref.: low) | | | | High (ref.: average) | | | |
| --- | --- | --- | --- | --- | --- | --- | --- | --- |
| Outcome measures | *b* | *p* | *LCI* | *UCI* | *b* | *p* | *LCI* | *UCI* |
| Social motivation |  |  |  |  |  |  |  |  |
| Belonging need | **-1.11** | **< .001** | -1.51 | -0.7 | **-0.90** | **< .001** | -1.30 | -0.50 |
| Reactions to belonging events | **-0.70** | **< .001** | -1.04 | -0.36 | **-0.61** | **< .001** | -0.95 | -0.28 |
| Person perception |  |  |  |  |  |  |  |  |
| Warmth | **-1.33** | **< .001** | -1.76 | -0.90 | **-0.55** | **0.012** | -0.98 | -0.12 |
| Competence | 0.04 | 0.857 | -0.35 | 0.42 | **0.55** | **0.002** | 0.21 | 0.88 |
| The Big Five |  |  |  |  |  |  |  |  |
| Extraversion | **-0.78** | **< .001** | -1.00 | -0.55 | **-0.82** | **< .001** | -1.03 | -0.61 |
| Agreeableness | -0.16 | 0.161 | -0.37 | 0.06 | 0.004 | 0.971 | -0.21 | 0.22 |
| Conscientiousness | -0.03 | 0.749 | -0.22 | 0.16 | -0.17 | 0.067 | -0.35 | 0.01 |
| Neuroticism | **-0.29** | **0.007** | -0.51 | -0.08 | **-0.26** | **0.014** | -0.46 | -0.05 |
| Openness | -0.12 | 0.271 | -0.33 | 0.09 | **0.24** | **0.020** | 0.04 | 0.44 |

*Note.* *b*s are unstandardized. Significant *b*s are in bold.

# Exploratory analysis: observer (participant) gender (Studies 3-5)

We analyzed responses from participants who identified as men or women (Study 3: n = 142; Study 4: n = 135; Study 5: n = 786). Using the same analytic approach reported in the main text, we added participants’ gender (reference: men) and the interaction term(s) between gender and the condition variable(s) as additional predictors to each regression model. To explore whether men and women hold different beliefs about solitude seeking others, we focus on the interaction effect between participant gender and target preference for solitude (high vs. low)—the effect we examined consistently across studies. The unstandardized regression coefficients are plotted in a Figure below. Of 30 interactions terms tested, only five were significant, none of which remained robust across the studies.

**Supplementary Figure 3**

The interaction effects between participant gender and target preference for solitude (high vs. low) in Studies 3-5


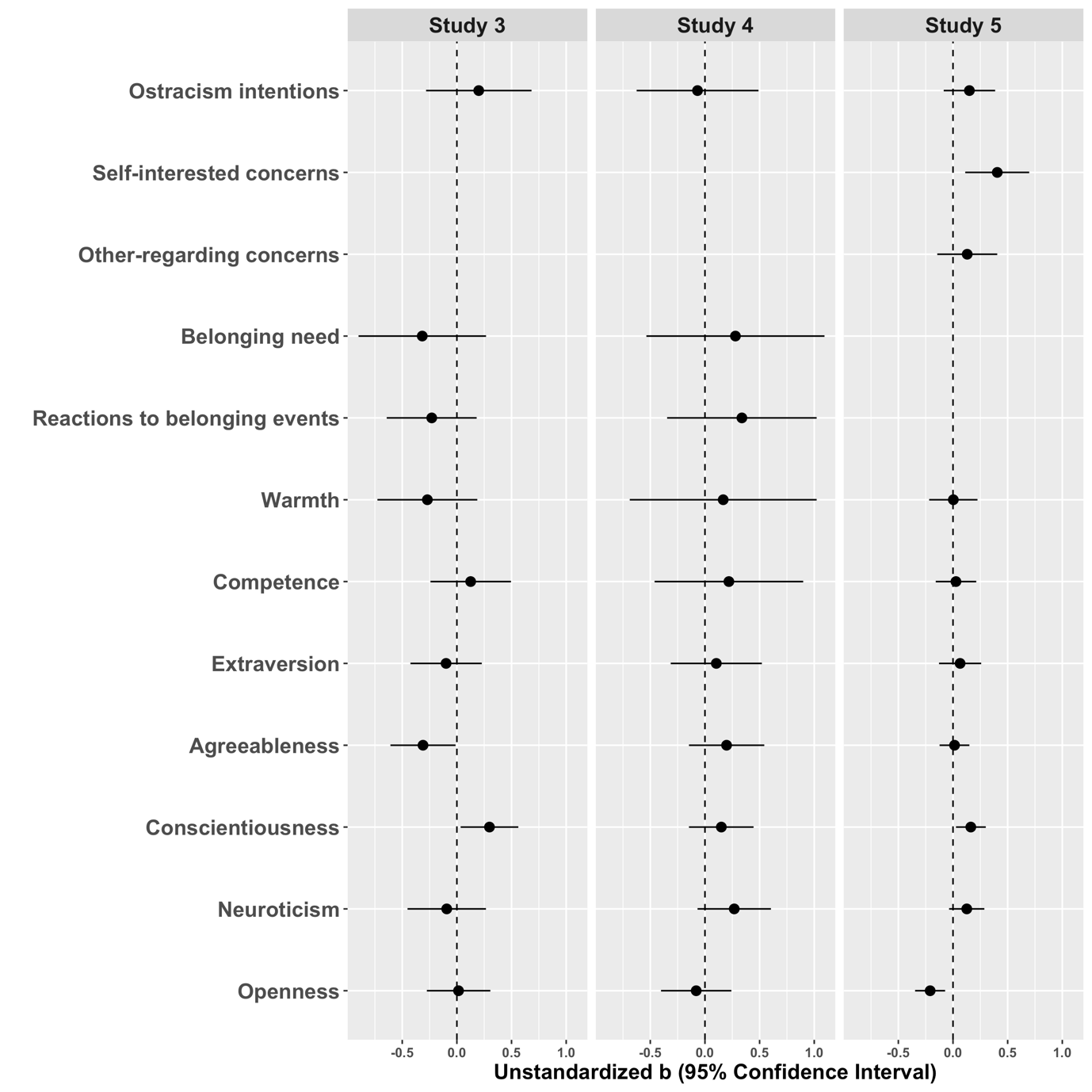

Supplement: Supplementary_Materials_2020_08_30 – Supplemental material for Leaving the Loners Alone: Dispositional Preference for Solitude Evokes Ostracism [file Supplementary_Materials_2020_08_30.docx]
